# Inferring neural sources from electroencephalography: foundations and frontiers

**DOI:** 10.1088/1741-2552/ae3e16

**Published:** 2026-02-13

**Authors:** A R Phillips, Y S Vakilna, D EPMoghaddam, A Banta, J C Mosher, B Aazhang

**Affiliations:** 1 Department of Electrical and Computer Engineering, Rice University, Houston, TX 77005, United States of America; 2 Texas Institute for Restorative Neurotechnologies (TIRN), Department of Neurology, McGovern Medical School, University of Texas Health Science Center at Houston, Houston, TX 77030, United States of America; 3 Center for Neural Systems Restoration (CNSR), Department of Neurosurgery, Houston Methodist Research Institute, Houston, TX 77030, United States of America

**Keywords:** source localization, stereo EEG (sEEG), EEG source imaging, high-density EEG, forward and inverse modeling, multimodal integration, neural signal processing

## Abstract

Electroencephalography (EEG) provides robust, cost-effective, and portable measurements of brain electrical activity. However, its spatial resolution is limited, constraining the localization and estimation of deep sources. Although methods exist to infer neural activity from scalp recordings, major challenges remain due to high dimensionality, temporal overlap among neural sources, and anatomical variability in head geometry. This topical review synthesizes inverse modeling approaches, with emphasis on nonlinear methods, multimodal integration, and high-density EEG systems that address these limitations. We also review the forward model and related background theory, summarize clinical applications, outline research directions, and identify available software tools and relevant publicly available datasets. Our goal is to help researchers understand traditional source estimation techniques and integrate advanced methods that may better capture the complexity of neurophysiological sources.

## 1. Introduction

As early as 1875, researchers have recorded electrical signals from living mammalian brains [1]. Fifty years later, in the 1920s, researchers began to record electroencephalography (EEG) from the human scalp, sparking decades of investigation and debate regarding the source of this signal [2]. By the 1970s experimental neurobiologists, theoretical biophysicists, clinical researchers, and electrical engineers were all actively contributing to the burgeoning field of EEG source analysis [3–7]. Theories emerged regarding a dipole model of EEG sources, where intracellular and extracellular flow of ions in pyramidal neurons create dipolar charges large enough to be measured at a distance [8]. However, the realities of winding gyri and sulci beneath heterogeneously conductive cranial bone present additional challenges to the already ill-posed problem of source estimation from EEG [9]. Regardless, it became clear that the EEG system of electrical sensors recording from the scalp, despite its challenges, offered a promising method to noninvasively infer underlying neurophysiological activity.

In the hundred years following this early pioneering work, researchers and clinicians have found numerous uses of the EEG signal. These uses include guiding neurologists and neurosurgeons to treat epilepsy, providing anesthesiologists measures of consciousness, assisting somnologists in sleep stage analysis, and offering cognitive scientists a glimpse into the stereotyped activity relating to perception and language processing [10–13]. The EEG signal has also been explored as a biomarker for numerous conditions, including depression, mild traumatic brain injury, and dementia [14–16]. At the same time, advances in techniques for source estimation from EEG have allowed for improved spatial resolution, scientific discovery, and clinical applications [17]. The development of high-density EEG (hd-EEG), which employs 64 or more channels, has helped overcome the spatial resolution limitations of standard EEG systems and improve the performance of source localization methods [18, 19]. EEG remains a popular choice for the development of noninvasive neurophysiological biomarkers and brain-computer interfaces due to its relatively low-cost and portable nature [20].

However, EEG is not the only modality for measuring neural activity. Invasive approaches such as stereotactic EEG (sEEG) involve the implantation of depth electrodes, enabling the recording of electrical activity closer to the sources. Other noninvasive alternatives leverage magnetic techniques to image neural activity, which often require cryogenic cooling and shielded environments, limiting their portability. Magnetoencephalography (MEG), for example, detects the magnetic fields generated by currents in the skull, offering a complementary signal to EEG due to the orthogonal orientation of the electric and magnetic field components. Other modalities contribute by modeling the biophysical structure of the subject’s head or by inferring neural activity indirectly through changes in blood flow and oxygenation, which are believed to reflect underlying neuronal activation. Magnetic resonance imaging (MRI), for example, although primarily used for structural imaging to help biophysical modeling of cranial geometries, can also capture functional activity of neural sources through techniques such as functional MRI (fMRI). Each modality offers distinct advantages and limitations in terms of spatial resolution, temporal resolution, invasiveness, and portability, but none of the methods are as portable or economical as EEG.

**Figure 1.**
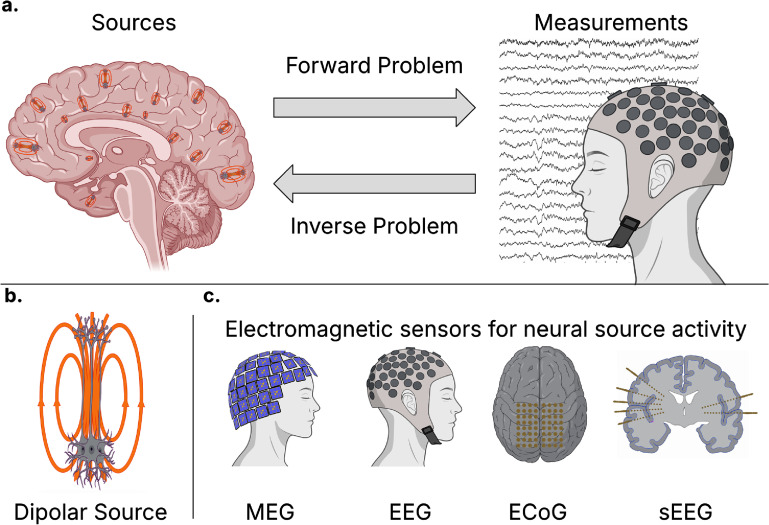
Overview of the forward and inverse problems in neural source estimation and associated recording modalities. (a) Schematic representation of the relationship between neural activity and sensor data. Here, the forward problem models how electromagnetic fields generated by distributed neuronal sources propagate through head tissues to be recorded as measurements at the sensors, and the inverse problem leverages these sensor measurements to estimate the location, orientation, and amplitude of the underlying sources in the brain, which may be time-varying. (b) Illustration of the dipolar model of neuronal activity, where ensembles of pyramidal neurons form equivalent dipole moments due to the flow of ions across the length of the cell. (c) Common electromagnetic sensing modalities used to record neural activity. These include noninvasive methods, such as magnetoencephalography (MEG) and electroencephalography (EEG), and invasive intracranial methods, including electrocorticography (ECoG) placed on the cortical surface and stereoelectroencephalography (sEEG) using penetrating depth electrodes.

This topical review synthesizes neural source estimation and localization techniques emphasizing EEG-based approaches. We begin by reviewing the background on neural sources and modalities to measure these sources before summarizing models that solve the forward problem of propagating source activity to sensor measurement recordings. We then review existing models to solve the inverse problem of estimating source activity from the sensor measurements. An illustration of the forward and inverse problem of neural source estimation and electromagnetic recording modalities is shown in figure 1. The review includes a summary of state-of-the-art methods, including techniques that model nonlinearities, scale to higher-density EEG recording systems, and leverage multiple modalities. Additionally, we discuss extensions of source estimations, such as estimating invasive measurements from noninvasive measurements. This discussion includes a summary of the key challenges remaining in neurophysiological source estimation. After reviewing techniques for source estimation, we synthesize the clinical uses of neural source estimation in practice today and potential future applications of neural source estimation and review the existing software and data available for researchers and clinicians to estimate source activity from EEG data. Finally, we propose a strategic roadmap for the field, outlining concrete milestones across varied time horizons to continue improving neural source estimation techniques. This work aims to offer a summary of the state-of-the-art techniques and challenges in neural source estimation, extending beyond linear inverse problem modeling on standard EEG systems.

## 2. Background

### 2.1. What are neural sources?

From a signal processing perspective, sources refer to the generators of signals. In other words, a source is the origin of information. In the context of the brain, the nature of neural sources has been debated [21, 22].

The dipole theory is a biophysical theory that relies on fundamental electromagnetic physics and volume conduction for the neuronal source to be measurable at a distance. The cellular origin of these dipolar sources is widely attributed to pyramidal neurons [23], whose aligned geometry and orientation within the cortex facilitate the summation of extracellular potentials and allow for spatially distributed current flows rather than a simple imbalance of charge. During synaptic activation, ionic flow into the cell at the distal dendrites and exit via other parts such as the soma or basal dendrites, creating a separation of charge and forming postsynaptic transmembrane currents [24]. As a result, the dipole theory of neurological sources models current sources or sinks within neuronal tissue via equivalent current dipole moments (illustrated in figure 1(b)), which are measured in Ampere-meters (A-m). The current of a single pyramidal neuron’s postsynaptic potential is typically modeled to be on the order of 20 fA·m, which suggests that evoked potentials observed in scalp recordings on the order of 10 nA·m comprise millions of synapses [25]. Biophysicists continue to add detail to this equivalent dipole model of neural sources, accounting for action potential quadrupoles, reversal potential, and presynaptic activity [26–28].

More detailed theories of neural sources expand beyond the notion of simple localized dipoles measurable at a distance via volume conduction. Simpler models of brain sources assume a limited number of dipoles in fixed locations, while more realistic models seek to estimate current sources distributed throughout the brain [29, 30]. Additionally, some neurophysiologists have preferred to view sources of measurable electrical brain activity as the result of cortical field potentials modulated by interactions with tissue [31]. The interaction of electrophysiological activity with tissue poses numerous challenges to the estimation of source activity. Volume conduction of the electrical activity along conductive pathways implicates changing dielectric properties of the brain tissue, cerebral fluids, and cranial bone [32–34]. Researchers in 2009 summarized 4 working source activity models: the equivalent-current dipole model, dipolar models in overdetermined problems, the cortical model, and the potential distribution inside the head [35]. The equivalent-current dipole model referred to the assumption of a single macro dipole, and the dipolar models in overdetermined problems referred to the models considering a small number of sources. The cortical model assumed no contribution of deep sources, while the potential distribution model generalizes across these models without making claims regarding the origins of these potential sources. Since that time, researchers have converged upon the distributed potentials resulting from micro and macro current sources and sinks forming dipoles or higher order n-poles [8, 36]. Additionally, research indicates that subcortical activity influences electrophysiological recordings of brain activity, especially with high density EEG [37] or MEG [38].

For many engineers and clinicians, the exact nature of neural sources is secondary to its utility as an underlying signal that can cause or explain neurological or psychiatric function and dysfunction. From the engineering perspective, the problem of estimating sources across the brain is underdetermined with multiple potential solutions, so biological, physical, or application-driven constraints are necessary to find purported sources of activity. As a result, researchers often take data-driven approaches, making use of functional connectivity and biophysical assumptions to estimate sources [39, 40]. Clinicians, especially in epilepsy monitoring units, focus on identifying functional regions, such as the epileptogenic zone and eloquent cortex [41, 42]. From these practical perspectives, the benefits of source estimation techniques are their ability to explain and treat neurophysiological function and dysfunction.

### 2.2. How are sources measured?

Neural source activity is measured via two main categories: direct electromagnetic recordings and indirect hemodynamic measurements. Electromagnetic methods offer high temporal resolution, while hemodynamic methods may provide greater spatial resolution, with ongoing efforts to integrate modalities. In the following sections, we offer a brief overview of the modalities available to infer neural source activity and highlight active areas of research focused on integrating multiple modalities.

### 2.3. Direct electromagnetic recodings

#### 2.3.1. Electroencephalography

EEG measures brain electrical activity as voltage differences between an electrode and a reference. Scalp EEG uses a standardized array to record scalp voltages against a non-neuronal reference, such as the mastoid bones or earlobe. Computational processing allows re-referencing and algorithms to estimate scalp-measurable source potentials. Source estimation algorithms may assume fixed [43, 44] or distributed sources [45, 46]. Key challenges include noise (e.g. motion artifacts, electromagnetic interference, heterogeneous propagation) and limited spatiotemporal resolution (depth and cortical). hd-EEG arrays improve spatial resolution [18, 19]. Approaches addressing these limitations include incorporating individual morphology via multimodal imaging [47, 48] and using complementary modalities like MEG and functional near-infrared spectroscopy (fNIRS) to improve source estimation quality and efficiency [49–53]. For reviews on the history and applications of EEG source localization, see [2, 14, 30, 54, 55].

#### 2.3.2. sEEG

sEEG measures extracellular electric potential inside the brain via neurosurgically inserted penetrating depth electrodes. This provides voltage measurements closer to the source, reducing attenuation and noise compared to scalp EEG. However, the procedure is invasive, requiring specialized neurosurgeons and equipment [56]. While a meta-analysis found complication rates of 0.9%–1.7% [57], a recent large cohort study reported zero complications [58]. The heterogeneous and subject-specific spatial distribution of sEEG contacts complicates inverse modeling and algorithm generalization, requiring precise morphological and contact locations to be accounted for [59]. Automated or semi-automated frameworks using pre-surgical MRI and post-implantation CT scans help localize these contacts [60–63]. The sEEG recordings serve as a complementary modality for data-driven scalp EEG source localization techniques [64–66]. Due to its invasive nature, sEEG is clinically restricted (e.g. drug-resistant epilepsy) and infeasible for source estimation in most populations [67]. Nonetheless, intracranial clinical and animal recordings offer a valuable research resource and potential training signal for source estimation models, and there is active research in identifying the shared information between scalp and intracranial EEG (iEEG) [68–70].

#### 2.3.3. Electrocorticography (ECoG)

ECoG, another form of iEEG, involves electrodes placed beneath the skull on the cortex surface, either subdurally or epidurally [71]. Used clinically since 1939, ECoG monitors epileptiform activity and guides resection surgery, especially for superficial regions [72, 73], and is also used to study network dysfunction in Parkinson’s disease [74, 75]. Many centers now prefer sEEG because it is less invasive, samples 3D cortical/subcortical structures, and has lower complication rates (e.g. hemorrhage, infection) [76]. ECoG (like sEEG) offers high temporal/spatial fidelity, avoiding skull-induced signal attenuation, but lacks sEEG’s 3D spatial coverage [77]. Several studies have demonstrated that incorporating ECoG signals into inverse modeling pipelines can improve source localization accuracy, especially when used to constrain or validate solutions [77–79]. As a result, similar benefits may be obtained by using sEEG data to constrain noninvasive source estimation techniques.

#### 2.3.4. Magnetoencephalography

MEG records neural activity from brain electrical currents using magnetometers to measure magnetic field changes and gradiometers to measure their spatial gradients. As a result, MEG measures aspects of the source electromagnetic field complementary to EEG. Magnetic fields generated by neural currents are also less sensitive to tissue and bone attenuation than electric field potentials. However, the interface between tissues of differing conductivities, such as the skull [80] cerebrospinal fluid (CSF) [81], can alter the distribution of secondary volumetric currents, which influence the measured field. This technology was first enabled by superconducting quantum interference devices, which require significant amounts of cooling that limits their portability [82]. In recent years, researchers have developed more portable magnetometers that function at ambient temperatures. These technologies include the spin-exchange-relaxation-free (SERF) optically pumped magnetometer (OPM) [83] and the magnetic-field-modulation-free OPM [84], the latter achieving broader bandwidth than the SERF OPM. Further advances in MEG portability will help expand its utility in source localization and estimation. We direct the reader to [25, 85] for further reading on MEG source estimation.

### 2.4. Functional hemodynamic measurements

MEG, along with scalp, stereo, and iEEG are the primary means to directly measure source electrical activity. However, several modalities measure the hemodynamic response, which is believed to correspond to source activity [86]. Although these measurements do not directly measure dipolar sources, functional measures of source activity have shown promise in localizing and estimating cortical and subcortical activity. Additionally, functional and indirect measurements have shown a complementary role in source estimation by capturing neurophysiological dynamics.

#### 2.4.1. fMRI

MRI uses magnetic fields and radio pulses to generate images of tissue. Structural MRI provides 3D brain images with resolutions from ∼0.2 mm at 11.7 T, to 0.5 mm at 7 T, and 1–2 mm at 3 T [87–90]. These high-resolution images can help constrain subject-specific biophysical source modeling and localize intracranial recording contacts. fMRI infers neuronal activity from blood-oxygen-level-dependent changes in blood flow [91]. While this method is noninvasive and offers strong spatial resolution, the temporal resolution is limited to seconds. Although some studies have reached sub-second sampling rates [92, 93], the seconds-long biological timescale of the hemodynamic response [94] remains a limiting factor for further technical advances. MRI systems usually require large, cooled magnets, but low-field portable scanners are emerging [95–97]. Multimodal integration of fMRI with EEG has shown improved source localization spatial resolution [48, 98, 99]. However, EEG systems are not always MRI compatible, and MRI compatible EEG signals are typically contaminated with artifacts despite ongoing artifact reduction efforts [100]. When feasible, fMRI remains a useful concurrent modality for estimating source activity.

#### 2.4.2. fNIRS

fNIRS uses the absorption of near-infrared light by oxygenated and deoxygenated hemoglobin to estimate hemodynamic activity. Since its first single-channel recordings in 1992, fNIRS has developed into multichannel systems capable of three-dimensional tomographic reconstructions [101]. High-density diffuse optical tomography (HD-DOT) helps extend the limited field of view, but imaging sensitivity remains limited beyond 1.5–2 cm beneath the skull [102]. Techniques such as confocal time-of-flight DOT have been shown to improve the resolution to millimeter scales [103]. The 2–10 Hz temporal resolution of fNIRS is faster than fMRI but slower than EEG [104]. As with fMRI, the biological timescale of the hemodynamic response constrains fNIRS analysis. Integrating fNIRS with electrical modalities, such as EEG, may offer complementary information that enhances functional network modeling and helps localize pathological source regions, including the epileptogenic zone [105–107]. Given its potential as a portable, noninvasive complement to scalp EEG, further research on synchronous EEG-fNIRS recordings for source estimation is needed.

#### 2.4.3. Other hemodynamic imaging modalities

While fNIRS and fMRI are the most commonly used noninvasive methods for measuring brain activity besides EEG, several other modalities are worth mentioning due to their role as complementary modalities. For example, emission tomography such as positron emission tomography (PET) and single-photon emission computed tomography (SPECT) use radiotracers to measure cerebrovascular activity [108]. These methods have limited spatial resolution or source estimation capability on their own, but demonstrate improved ability to determine diagnostic outcomes when combined with EEG source imaging [109, 110], although the expensive and non-portable nature of emission tomography limit their widespread use.

Other modalities remain in earlier stages of development for functional source imaging. Functional ultrasound (fUS) leverages the Doppler effect and ultrasound waves to image fluid flow. While this method is useful for measuring blood flow velocity in major arteries, it has also been used to image neuronal hemodynamic responses in humans [111, 112] and efforts are underway to integrate EEG with fUS [113]. Photoacoustic tomography (PAT) is an emerging technology that also leverages ultrasonic principles by pulsing laser light which is absorbed by the tissue and causes thermoelastic expansion that generates ultrasound waves [114]. PAT achieves greater penetration depth for imaging than fNIRS [115] and has been used to measure BOLD signal in humans [116], but further work is needed to determine proper molecular targets [117] and develop algorithms that incorporate EEG and estimate source activity. Another promising noninvasive modality is electromagnetic wave-based imaging (EMI). Gigahertz frequency waves have been used to estimate varying dielectric properties of tissue, including the brain [118–120], where researchers are investigating the detection and localization of stroke [121, 122]. Because the dielectric properties are expected to change not only with changes in blood flow but with ionic concentration changes caused by action potentials, there are efforts to estimate neuronal firing with EMI [123, 124], but further work is needed to ensure the focality of the measurement and maintain specific absorption radiation rates within the safe limits.

To improve source EEG and MEG localization and estimation, promising complementary modalities could help determine conductivity for personalized modeling. Electrical impedance tomography (EIT), for example measures tissue conductivity using small currents and can track intracranial conductivity changes related to blood flow and pressure [125, 126]. It has clinical applications [127, 128], complements EEG/MEG with patient-specific conductivity models [129], and can even image neuronal activity at high spatiotemporal resolution in peripheral nerves [130, 131]. Integrating EIT with OPMs is an emerging approach for fast, noninvasive source imaging [132]. An even more nascent conductivity measuring method is magnetoacoustic tomography (MAT), where a time-varying external magnetic field generates acoustic fields which can estimate the conductivity distribution of tissue [133]. Research efforts continue to be made to develop MAT systems and characterize their biophysical responses, especially as a technique for electrical impedance imaging [134, 135]. Modalities that measure head conductivity need more research to validate their measurements and to build models that can properly use that data.

These modalities each represent promising directions for noninvasive imaging of neural activity, but further work is needed to develop the methodologies, validate their biological interpretation, and integrate these techniques with modalities, such as EEG and MEG, that directly measure electrical activity in the brain.

## 3. Forward models

A core challenge in neural source analysis is accurately modeling the forward propagation of source activity to scalp sensor measurements. This section reviews established approaches to the forward problem, from simplified models to more advanced methods that account for individual anatomical variability, and highlights promising directions for improving both model accuracy and computational efficiency. In EEG source analysis, the forward problem plays two key roles: it provides a biophysically grounded framework for validating source estimates and enables the generation of realistic simulations of brain activity and corresponding EEG data.

More broadly, estimating neural source activity typically involves three major steps: 1. modeling neural electrical activity, 2. modeling head volume conduction to relate neural sources to scalp potentials (the EEG forward problem), and 3. reconstructing source activity from EEG measurements (the inverse problem) [136, 137]. The forward problem predicts electric field measurements on the scalp given source configurations, while the inverse problem attempts to estimate the sources that gave rise to the recorded signals. The forward problem can be described as finding function *$g$* (1), while the inverse problem seeks the function *f* (2). Here, $\mathbf{S}\in\mathbb{R}^{p\times T}$ denotes the time-varying source signal across *p* locations and *T* time steps while $\mathbf{M}\in\mathbb{R}^{N_m\times T}$ denotes the signal measurements across *N_m_* sensors or channels. Typically, *N_m_* is between 16 and 32 channels for standard EEG or 128–256 for high density EEG, although there is evidence that as few as 6 channels may be capable of estimating a single source [138] and ultra high density EEG systems with 1024 channels are an active area of research [139],

\begin{align*} g: &amp; \ \mathbf{S} \mapsto \mathbf{M}\end{align*}

\begin{align*} f: &amp; \ \mathbf{M} \mapsto \mathbf{S}.\end{align*}

The accuracy of the forward model directly influences the precision of source localization and therefore plays a critical role in solving the EEG inverse problem. Traditional EEG forward models typically assume linearity, static conductivity, and isotropic tissue properties, simplifications that limit the physiological realism of the solutions [136]. Recent studies highlight the importance of incorporating more accurate, nonlinear forward models that capture biophysical complexity and individual anatomical variability, including morphological variation caused by developmental or pathological changes [140–142]. In this section, we approach the EEG forward problem by first reviewing models of neural electrical activity, including a mathematical framework, followed by an overview of the most commonly used head volume conduction models.

### 3.1. Neural mass models ((NMMs) and generative models of EEG

While foundational models like the Hodgkin–Huxley formalism established the mathematics of single-neuron membrane dynamics [143], these microscopic descriptions do not scale to macroscopic neuroimaging. The Wilson–Cowan model bridged this gap by formally describing the activity of interacting neural populations [144]. However, interpreting EEG requires linking these population dynamics directly to the biophysics of signal generation, which the Jansen–Rit framework addressed by modeling the intrinsic connectivity between pyramidal cells and interneurons within a cortical column [145, 146]. Crucially, this connects mesoscopic dynamics to the EEG forward problem: the summed post-synaptic potentials of perpendicularly oriented pyramidal cells yield a time-varying equivalent current dipole. This dipole serves as the biophysical source for forward modeling of the scalp potentials, connecting neural mass equations to observable EEG signals.

### 3.2. Forward modeling: physical approximations

To mathematically solve for scalp potentials, the quasi-static approximation is applied, since the low-frequency nature of EEG renders displacement currents and wave propagation within the head volume negligible. In a linear ohmic medium, the electric field is proportional to the current density and satisfies $\mathbf{E}(\mathbf{r}) = -\nabla\phi(\mathbf{r})$, where $\phi(\mathbf{r})$ is the electric scalar potential at location **r**. Charge conservation reduces Maxwell’s equations to the Poisson-type partial differential equation

\begin{align*} \nabla \cdot \left(\sigma\left(\mathbf{r}\right)\,\nabla \phi\left(\mathbf{r}\right)\right) \; = \; \nabla \cdot \mathbf{J}_\mathrm{p}\left(\mathbf{r}\right),\end{align*}

where $\sigma(\mathbf{r})$ denotes spatially-varying conductivity and $\mathbf{J}_\mathrm{p}$ is the primary current density caused by source activity. Under the quasi-static approximation, (3) is linear in the source term. Consequently, the potential *φ* generated by a single dipole scales linearly with its dipole moment, and the contributions from multiple dipoles combine via electromagnetic superposition to produce the measurable scalp potentials.

*Sensor-domain mapping.* In practice, researchers evaluate (3) at discrete scalp sensor locations $\{\mathbf{r}_m\}$. A single current dipole is the primary current density $\mathbf{J}_\mathrm{p}$ evaluated at a singular point **r**_*q*_, with corresponding dipole moment ***q***. The potential at sensor *m* is denoted for this dipole as

\begin{align*} \phi\left(\mathbf{r}_m\right) \; = \; \mathbf{g}\left(\mathbf{r}_m,\mathbf{r}_q\right) \cdot \mathbf{q},\end{align*}

where, $\mathbf{g}(\mathbf{r}_m,\mathbf{r}_q)$ is the sensor-specific lead-field vector describing the linear relationship between the unit dipole and measured electrical potential. For clarity, we adopt a theoretical ‘infinite’ reference in this notation, while in practice, recordings use a physical reference; results stated under the ‘infinite’ reference can be re-referenced in the usual way without changing the underlying physics. For multiple dipoles indexed by *k*, by electromagnetic superposition, the potential is simply the sum

\begin{align*} \phi\left(\mathbf{r}_m\right) \; = \; \sum_k \mathbf{g}\left(\mathbf{r}_m,\mathbf{r}_{q_k}\right) \cdot \mathbf{q}_k.\end{align*}

For convenience, we include table 1 describing the key mathematical notations used to synthesize the forward and inverse models.

**Table 1.** Summary of the notation used to describe the electromagnetic forward and inverse problems, including scalars, vectors, matrices, and functions.

Symbol	Type/dim.	Description
** $\mathbf{S}$ **	$\mathbb{R}^{p \times T}$	Source amplitude matrix
** $\mathbf{M}$ **	$\mathbb{R}^{N_m \times T}$	Measurement data matrix
** $\mathbf{N}$ **	$\mathbb{R}^{N_m \times T}$	Noise matrix

$N_m, p$	$\mathbb{Z}^+$	Number of sensors and sources
** $\mathbf{A}$ **	$\mathbb{R}^{N_m \times p}$	Forward-field matrix for multiple dipoles
*g*	Function	Forward mapping: $\mathbf{S} \mapsto \mathbf{M}$
*f*	Function	Inverse mapping: $\mathbf{M} \mapsto \mathbf{S}$

$\phi(\mathbf{r})$	$\mathbb{R}$	Scalar electric potential
**r**	$\mathbb{R}^3$	Spatial position vectors (dipoles: **r**_*q*_, sensors: **r**_*m*_)
$\sigma(\mathbf{r})$	Scalar or $3\times 3$	Electrical conductivity, isotropic or anisotropic
$\mathbf{q}(t)$	$\mathbb{R}^3$	Dipole moment vector at time *t*
$\boldsymbol{U}(\mathbf{r}_q)$	$\mathbb{R}^3$	Dipole orientation (assumed normal to cortex)
$\mathbf{m}(t)$	$\mathbb{R}^{N_m}$	Sensor data vector at time *t*
$\mathbf{n}(t)$	$\mathbb{R}^{N_m}$	Noise vector at time *t*
$\mathbf{g}(\mathbf{r}_m,\mathbf{r}_q)$	$\mathbb{R}^3$	Lead-field vector for dipole *q* and sensor *m*
$\mathbf{a}(\mathbf{r}_q)$	$\mathbb{R}^{N_m}$	Forward field vector for unit dipole at **r**_*q*_

### 3.3. Head volume conduction models for EEG forward problem

Once neural electrical activity is modeled, typically using current dipole sources as described above, the next step in solving the EEG forward problem is to define a head volume conduction model. This model represents the conductive properties of head tissues and describes how electric currents propagate from neural sources through various tissue compartments to the scalp, enabling the computation of the resulting surface potentials.

Early approaches to EEG forward modeling employed simplified geometries, such as the homogeneous spherical head model, and later, multi-shell concentric spherical models, which allowed for analytical or semi-analytical solutions to the Poisson equation (3) governing electric potential distribution [147, 148].

While computationally efficient, these models do not capture the complex and heterogeneous structure of the human head. A growing body of research has demonstrated that factors such as skull thickness, tissue curvature, and sharp conductivity discontinuities significantly impact the accuracy of EEG forward modeling [149–151]. Consequently, the field has advanced toward realistic head models, constructed from high-resolution MRI segmentations that accurately delineate the geometry and conductivity profiles of major tissue types, including the scalp, skull, CSF, gray matter, and white matter [43, 152, 153].

**Figure 2.**
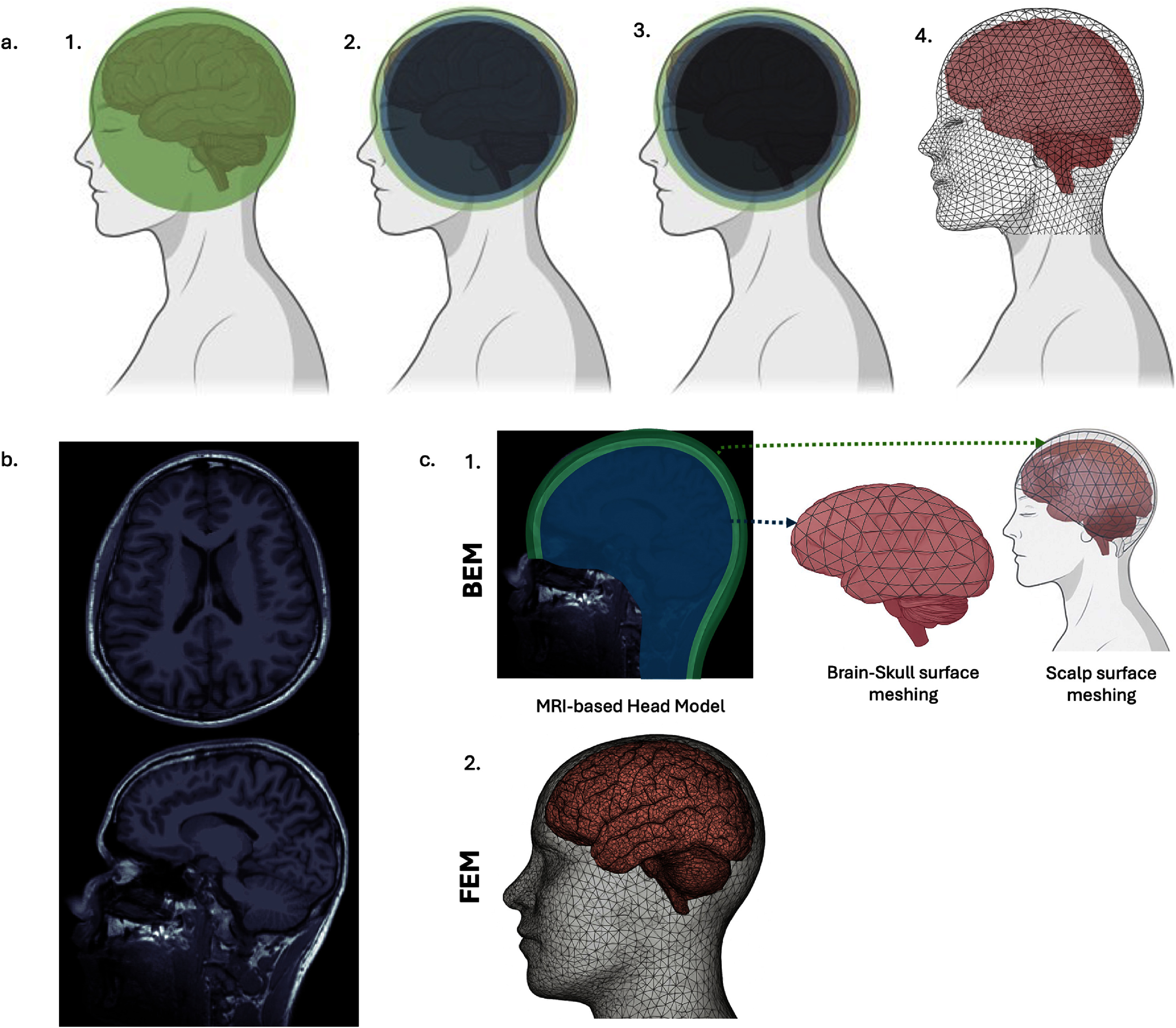
Overview of head modeling techniques for forward solutions. (a) Progression from simplified spherical approximations to realistic geometries. 1. Single-sphere model representing the head as a homogeneous conductor. 2. Three-shell concentric spherical model differentiating the scalp (green), skull (blue), and brain (navy). 3. Four-layer spherical model incorporating cerebrospinal fluid (CSF) (navy) between the skull and brain (dark gray). while additional layers may capture other tissue properties. 4. Realistic head model generated from anatomical data using surface tessellation. (b) Structural magnetic resonance imaging (MRI) scans (axial and sagittal views) utilized to define individual anatomy for realistic modeling. Examples courtesy of Brainstorm [195]. (c) Comparison of numerical methods for realistic head modeling based on MRI segmentation. 1. The boundary element method (BEM) models the head as a set of nested, closed surfaces (e.g. brain–skull, skull–scalp, and scalp surfaces) using 2D triangular meshes. BEM assumes piecewise homogeneous and isotropic conductivity within each compartment. 2. The finite element method (FEM) discretizes the entire head volume into small 3D elements (such as tetrahedra or hexahedra). This volumetric approach enables detailed modeling of complex geometries and local tissue properties, including inhomogeneous and anisotropic conductivities.

Due to the anatomical complexity of these realistic models, strictly analytical solutions are not feasible. As a result, numerical methods such as the boundary element method (BEM) and finite element method (FEM), along with historically used methods such as finite difference method (FDM), and, less commonly, finite volume methods (FVMs) have been used to solve the EEG forward problem in realistic head geometries [152, 154, 196]. These methods and their variants and extensions each offer advantages and limitations in terms of anatomical fidelity, numerical stability, computational efficiency, and ease of implementation. These trade-offs are explored in the following subsections, with an illustration of the forward modeling techniques presented in figure 2.

*Importance of anatomical detail and compartmentalization.* Modern EEG forward modeling relies on anatomically detailed, subject-specific head models constructed from high-resolution MRI scans. These models typically include at least five tissue compartments: scalp, skull, CSF, gray matter, and white matter. More detailed models range from six-compartment versions that distinguish between compacta and spongiosa skull tissues [155, 156] to more complex frameworks incorporating as many as 12 distinct tissue types [157, 158]. Several studies have shown that inaccurate anatomical representations, especially omitting CSF or failing to differentiate gray and white matter can significantly distort scalp potential distributions and reduce source-estimation accuracy [155, 159–162]. For example, including the highly conductive CSF compartment has been shown to strongly influence signal topography and improve localization precision [161]. FEM-based approaches are well suited to represent this level of anatomical detail, whereas traditional BEM models are typically limited to 3–4 layers and assume homogeneous, isotropic conductivity within each shell.

The skull in particular poses significant challenges in developing realistic head models. A common assumption of the skull as a homogeneous isotropic shell often fails to capture significant anatomical nonuniformities. Structurally, the cranium is composed of three-layers: a conductive spongiform layer between highly resistive inner and outer compact bone table, each layer of inhomogeneous thickness [156, 163]. Furthermore, skull sutures, the fibrous joints connecting bone plates, introduce local high-conductivity paths that can act as electrical shunts [164]. Neglecting the distribution of spongiform bone and the specific geometry of sutures in the forward model can distort the topography of scalp potentials. In the context of the inverse problem, these modeling inaccuracies propagate as localization errors, particularly for superficial sources located near suture lines, thereby emphasizing the importance of realistic, subject-specific head models when source estimation accuracy is critical [141, 165].

Further, the accuracy of forward models depends not only on the geometry but also on the conductivity values assigned to each tissue. Skull conductivity is especially critical due to its low value and high variability across individuals [166]. Literature estimates for compact bone range from 5 to 10 mS m^−1^ and for spongiform from 16 to 40 mS m^−1^ [167], yet most simplified models assign a single homogenized value [156]. These assumptions can shift source localization by several centimeters [159, 168]. To address some of the challenges estimating the tissue conductivity, several data-driven methods for conductivity estimation have been proposed [169–171]. The authors of [172] compiled the reported conductivity metrics of various tissue, demonstrating wide ranges of values that support the need for complementary modalities that measure the individual’s tissue conductivity. Accurate modeling of conductivity remains a key frontier in improving the biophysical realism of EEG simulations.

#### 3.3.1. Spherical head models

The earliest approaches to EEG forward modeling employed simplified geometries, starting with the homogeneous sphere [147] and evolving into the three-shell concentric spherical model representing the brain, skull, and scalp [148]. While these formulations allowed for efficient semi-analytical solutions to Poisson’s equation (3), they fail to capture critical anatomical features, particularly the irregular shape and the spatially varying thickness and curvature of the skull, which can substantially impact the accuracy of EEG forward solutions [149–151, 173–176]. The recognition of these geometric limitations drove the initial development of realistic head models [43, 152, 177], though the computational cost of early numerical solvers spurred further attempts to refine analytical spherical frameworks.

To improve accuracy without abandoning analytical tractability, intermediate solutions were proposed, such as sensor-fitted spheres that optimize local geometry for each electrode [178], and four-layer models that incorporate CSF [179–181]. The inclusion of the CSF layer is particularly relevant as its high conductivity acts as a shunt for volume currents, strongly influencing the distribution of scalp potentials [182]. However, further efforts to increase the neuroanatomical included additional compartments in non-spherical geometries, necessitating non-analytical computational approaches. Consequently, modern EEG analysis has largely shifted toward the numerical solvers (BEM and FEM) discussed in the following sections, which can naturally handle complex, non-spherical geometries.

#### 3.3.2. Realistic head models

Early pioneering work, such as that by He *et al* [43], introduced the use of realistic head geometries derived from structural MRI, combined with numerical methods like the BEM, to replace idealized spherical models [43]. As modeling techniques advanced, subsequent studies incorporated greater anatomical and physiological detail, including more accurate representation of tissue boundaries and tissue anisotropy [183, 184].

Modern realistic head models are subject-specific, anatomically detailed representations of the human head, typically constructed from high-resolution structural MRI scans. These models include multiple tissue compartments, commonly the scalp, skull, CSF, gray matter, and white matter, with some models also incorporating additional structures such as the ventricles, cerebellum, and brainstem [161]. Each compartment is assigned a conductivity value based on experimental measurements or literature-derived estimates [168, 172, 185].

To address scenarios where individual MRI data are unavailable, researchers also adopted mean head models; population-averaged templates that provide a standardized yet less personalized alternative [186]. While these models lack the subject-specific precision of MRI-based geometries, they remain widely used in both research and clinical applications due to their accessibility and consistency [187, 188]. Given the anatomical complexity and the absence of analytical solutions for realistic head models, numerical methods have become indispensable tools for solving the EEG forward problem.

#### 3.3.3. Numerical methods for realistic EEG modeling

Realistic head models exhibit irregular geometries and complex conductivity patterns across tissue interfaces, rendering analytical solutions to the EEG forward problem infeasible. Instead, numerical methods are required to approximate the electric potential field generated by neural sources. Three principal numerical techniques are employed: the FDM the BEM, and the FEM.

FDM uses a regular voxel grid aligned with MRI data but is less flexible in handling curved and non-conformal surfaces. BEM models the head as a set of nested, piecewise-homogeneous compartments and computes solutions on tissue boundaries, offering computational efficiency in layered, isotropic media. FEM discretizes the entire head volume and is well-suited for incorporating anisotropic conductivities and complex geometries, albeit at a higher computational cost. Each method transforms the continuous partial differential equation governing electric potential (typically Poisson’s equation) into a solvable algebraic system. The choice of method depends on the trade-off between modeling accuracy, computational cost, and the specific demands of the EEG analysis.

FDM:

The finite difference method is one of the earliest numerical techniques for solving partial differential equations, relying on finite difference approximations over a structured Cartesian grid. In the context of EEG forward modeling, FDM discretizes the Poisson equation over a structured Cartesian grid, typically aligned with voxel data from structural MRI scans [150,154]. This approach allows for straightforward implementation and efficient computation, especially for simple geometries.

A major challenge in this context is the presence of strongly discontinuous conductivities at tissue interfaces (e.g. brain–CSF, CSF–skull), which standard FDM schemes (assuming smooth coefficients) fail to handle accurately. These limitations lead to significant errors, particularly at grid nodes that intersect multiple tissue types. To address this, several studies have proposed specialized schemes. For instance, Hédou-Rouillier [196] developed and analyzed a set of three-dimensional FDM schemes specifically designed to handle conductivity discontinuities at tissue interfaces.

Although FDM is generally less accurate than FEM or BEM, mainly due to its limited ability to capture complex anatomical geometry, it remains useful in scenarios that benefit from rapid computation, direct voxel-based modeling, or minimal preprocessing. Nonetheless, its role in modern EEG source localization has declined in favor of more flexible and biophysically detailed methods like FEM and BEM.

**FVM**: Finite volume methods discretize the forward problem by integrating the governing equations over small control volumes, thereby enforcing local current conservation. In EEG applications, FVM typically employs a structured grid of cubic voxels, with piecewise-constant conductivities defined at voxel centers and electric potentials defined at the nodal vertices. Although FVM and hybrid FVM–BEM formulations have been applied to realistic and anisotropic head models [189], their use has remained far less common than FEM or BEM and is now largely of historical or methodological interest in EEG forward modeling.

**BEM:** The boundary element method is a numerical technique used to compute the electric potentials on the scalp surface generated by current sources within the head [43, 152]. It models the head as a piecewise homogeneous and isotropic volume conductor, typically considering three nested surfaces: the brain–skull interface, the skull–scalp interface, and the outer scalp surface [152]. Each surface is tessellated with small two-dimensional, usually triangular, elements and surface integrals are approximated using basis functions such as constant or linear potential.

A known challenge in BEM is that the electric potentials are defined only up to an additive constant, resulting in a non-unique solution. This ambiguity can be resolved through techniques such as deflation [136, 190, 191]. Deflation works by constraining the solution space, typically by fixing the potential at one node or enforcing that the mean potential over all nodes is zero, thereby eliminating the arbitrary constant. Another issue arises from the large conductivity discontinuity near the skull (i.e. the conductivity ratio between the skull and brain tissues) which can introduce numerical instabilities. This is mitigated using the isolated problem approach (IPA), which improves numerical accuracy by isolating the inner skull surface during the initial solution [152]. In this implementation, the IPA is generalized to allow for additional layers within the modified boundary defined by the inner skull.

To overcome BEM’s computational demands, several optimized variants have been developed [192–194]. For instance, accelerated BEM variants improve efficiency by computing potentials only at electrode locations [192]. A more recent advancement is the BEM accelerated by the fast multipole method (BEM-FMM), introduced by Wartman *et al* [194], which achieves accurate forward solutions on high-resolution head models in under 90 s, making it feasible for large-scale or time-sensitive EEG/MEG applications.

**FEM:** Unlike BEM, which assumes piecewise homogeneous and isotropic compartments, the FEM offers a powerful numerical framework for solving the EEG forward problem with high anatomical and physical fidelity [184]. FEM discretizes the head volume into small elements, typically tetrahedra or hexahedra, and approximates the governing partial differential equations locally. This approach naturally accommodates complex anatomical geometries, spatially varying and anisotropic conductivities, and the inclusion of lesions or non-nested tissue interfaces. The impact of incorporating greater anatomical and electrical realism has been demonstrated in several studies examining both forward and inverse EEG solutions [161, 176]. Notably, Marin *et al* concluded that for robust and accurate min-norm imaging with EEG, it is essential to employ realistic head models that incorporate tissue anisotropy [176].

Historically, FEM was considered computationally demanding due to the need for volumetric meshes and large system matrices [197]. However, these concerns have been largely mitigated by substantial methodological and hardware advances. Modern mesh generators and GPU-accelerated implementations now enable high-resolution, subject-specific FEM simulations to be performed with efficiency previously reserved for simpler models [198–200]. With current software [201, 202], generating FEM meshes that perform comparable to standard BEM configurations is straightforward, although pushing toward higher anatomical fidelity introduces additional computational complexity. As a result, FEM has become widely regarded as a gold standard for realistic head modeling in EEG source analysis, while BEM remains an efficient alternative for simpler or layered head models [161].

To further improve accuracy and scalability, recent advances have introduced hybrid and specialized FEM-based methods aimed at enhancing anatomical fidelity and numerical efficiency [154, 203]. One such direction involves BEM–FEM hybrid methods, which combine the surface-based efficiency of the BEM with the volumetric flexibility of the FEM, enabling realistic head modeling while reducing computational cost [204]. Other FEM-based extensions, such as the Discontinuous Galerkin FEM, provide high-order accuracy and improved numerical stability, particularly for problems involving sharp conductivity transitions across tissue interfaces [154].

A recent promising extension is the Cut FEM (CutFEM), which embeds complex or evolving geometries (such as curved cortical surfaces or non-nested lesions) into a fixed background mesh [205, 206]. Rather than requiring a conforming mesh that exactly aligns with tissue boundaries, CutFEM permits the domain to be ‘cut’ from a structured or unfitted mesh. This approach eliminates the need for labor-intensive remeshing and supports high-fidelity simulations on anatomically realistic head models [206].

Recent advances in scientific computing have introduced deep learning surrogates and reduced-order models that approximate FEM solutions with significantly reduced computational cost [207, 208]. While their application in EEG forward modeling is still emerging, such models hold promise for enabling near real-time predictions in time-sensitive settings like closed-loop neurofeedback and brain–computer interfaces. Parallel developments in GPU-accelerated solvers and frameworks are also expanding the scalability of high-resolution simulations. Collectively, these innovations enable new generation of EEG forward modeling tools that balance anatomical fidelity, numerical robustness, and computational speed.

### 3.4. Advanced source models and spatial extent

The established EEG forward modeling techniques discussed represent neural sources as equivalent current dipoles. However, when the true generator is spatially extended or composed of complex current distributions, a single dipole can misrepresent both amplitude and spatial extent of the field [21, 36]. To address this, researchers have developed multipole expansions, including quadrupolar models, that capture higher-order spatial features of neural generators [101, 209]. For example, recent FEM-based frameworks allow efficient modeling of quadrupolar sources, which also improved the accuracy and stability for dipolar sources [209]. While distributed dipolar source models remain a widespread choice in source estimation, multipole forward models offer a complementary strategy that can capture higher-order spatial structure without requiring a large explicit dipole set. Although multipolar formulations are primarily developed in MEG forward and inverse modeling [210, 211] due to the rapid decay of the electric field, recent work indicates potential benefits for EEG forward modeling as well.

### 3.5. Forward model conclusions

This section summarized the development of the forward model for EEG source analysis, which predicts scalp measurements from brain activity. The forward model has evolved from simple spherical shell approximations to anatomically detailed, multi-compartment FEM models informed by subject-specific morphology, conductivity calibration, and in some cases, advanced source models such as multipolar expansions. However, simpler models maintain roles depending on the computational demands of the modeling task. More recent advances in advanced numerical methods like FEM provide higher anatomical and physiological accuracy. Methodologies to validate forward modeling is a critical field of research as well, with some researchers proposing intracranial electrical stimulation and synchronous EEG recording [19, 212]. Future directions aim to improve computational efficiency through hybrid methods, deep learning surrogates, and specialized algorithms to enable real-time, high-fidelity source analysis.

## 4. Inverse models

While forward models map source activity to sensor measurements, inverse models map sensor measurements back to source activity. In relation to (2), inverse models approximate *f* to identify sources **S** from measurements **M**. The following sections synthesize decades of research on forward modeling, presenting a unified mathematical framework for many of these linear models, identifying the assumptions and applications of these methods. After introducing these linear frameworks, we discuss methods that use advanced machine learning techniques to estimate source activity in a supervised manner. We conclude with a discussion of future research directions, including approaches that leverage simultaneous intracranial and scalp recordings.

### 4.1. Review of linear models

Electromagnetic source localization seeks to pinpoint the brain regions generating electrical activity measured on the scalp. Although the EEG and MEG measure different characteristics of the underlying source, the mathematical approach to estimate the source activity is fundamentally equivalent. This inverse problem is notoriously ill-posed; as a result, several advanced signal processing algorithms have been proposed over the past 40 years to reconstruct a plausible and verifiable inverse solution, once the head model is computed. These ideas build on Hämäläinen and Ilmoniemi [215] and Sarvas [216], who laid the mathematical foundations.

**Figure 3.**
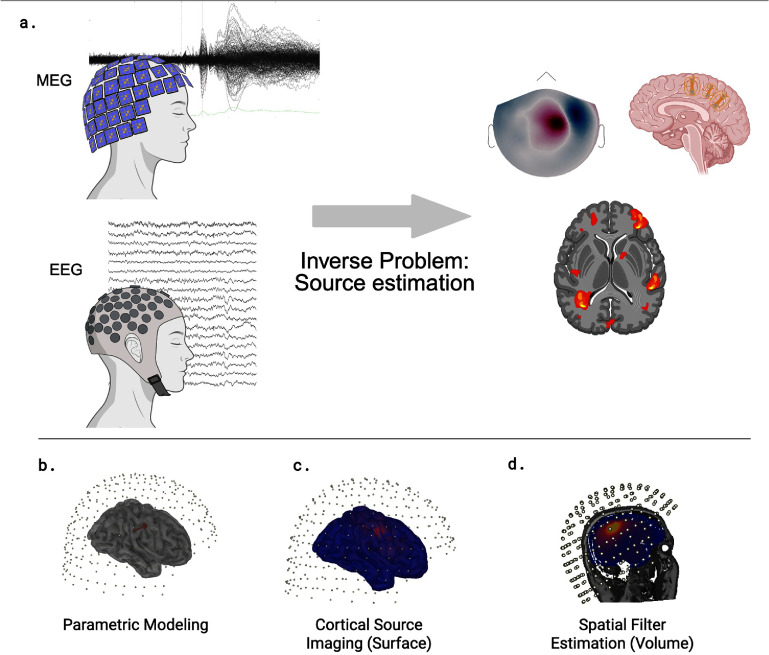
The inverse problem of neuronal source estimation and taxonomies of modeling approaches. (a) Overview of the inverse process. Noninvasive electromagnetic measurements obtained via magnetoencephalography (MEG) and electroencephalography (EEG) are processed to infer the underlying neural source activity. The results can be visualized as topographical scalp maps (right, top left), equivalent dipoles (right, top right), or distributed volumetric activity (right, bottom). (b) **Parametric modeling:** this approach assumes a small number of focal sources. The visualization shows an equivalent current dipole (ECD) reconstruction, where the location and moment of a source are estimated using least-squares fitting. (c) **Cortical source imaging (Surface):** a distributed inverse approach where sources are constrained to the cortical surface geometry. The example displays a current-density reconstruction computed using dynamic statistical parametric mapping (dSPM) [213]. (d) **Spatial filter estimation (Volume):** a volumetric approach that scans the brain using a grid of locations rather than a surface mesh. The example shows a source map computed using the neural activity index (NAI), a beamforming metric [214]. Panels (b)–(d) were generated with Brainstorm [195].

In the following sections, we follow a commonly used three-way taxonomy of linear methods, where figure 3 illustrates the inverse problem and example modeling approaches:

(i) **Current-density reconstruction**: Current-density reconstruction models represent the cortex using a dense array of fixed candidate dipoles. Because the dipole locations are fixed, the inverse problem simplifies to a linear estimation of the dipole amplitudes. However, since the number of unknown source amplitudes vastly exceeds the number of sensors, this inverse problem is severely underdetermined, necessitating the use of regularization or priors to constrain the solution.
(ii) **ECD reconstruction**: ECD reconstruction assumes that only a small number of focal sources are active in the brain, each modeled as an ECD with unknown parameters. The inverse problem is then cast as a non-linear optimization to find the best-fitting dipole parameters for a prespecified number of dipoles, which yields a discrete solution that explains the data.
(iii) **Spatial filtering**: Spatial filters, including adaptive beamformers, construct linear weights to pass activity from a target location while suppressing interference from elsewhere. These methods, such as the linearly constrained minimum variance (LCMV) beamformer, generate power or signal-to-noise ratio (SNR) maps that can reveal multiple concurrent sources without prespecifying their number [214].

The source-space definition represents the foundational modeling decision in practice. A poor choice exacerbates issues like depth bias, spatial leakage, and false positives, whereas adopting anatomically constrained spaces enhances both conditioning and interpretability. A geometry-agnostic strategy distributes candidate dipoles on a dense volumetric grid inside the head, akin to tomographic sampling [24]. For healthy participants, dominant EEG generators arise from aligned cortical pyramidal populations, motivating a cortically constrained source space with surface-normal orientations [36, 137, 217]. After MRI segmentation, the gray-white boundary is tessellated into a triangular mesh; a unit dipole is placed at each vertex and oriented along the local surface normal, reflecting apical dendrites orthogonal to the cortex. Capturing gyral and sulcal geometry at millimeter resolution typically requires 10^4^–10^5^ such elements. In clinical populations, especially epilepsy, generators can be deep or noncortical and may involve dysplastic cortex; analyses should therefore consider volumetric source spaces, relaxed orientation constraints, and explicit subcortical compartments when indicated by hypotheses.

*Algebraic formulation of the inverse problem.* The approach to mathematically modeling the inverse problem builds off the algebraic model of the forward problem, which we synthesize in the following sections. Consider a dipole at location **r**_*q*_ with fixed unit orientation $\boldsymbol{U}(\mathbf{r}_q)$. At time point *t*, let the dipole moment be $\mathbf{q}(t) = \boldsymbol{U}(\mathbf{r}_q)\cdot s(t)$, where *s*(*t*) is the scalar dipole amplitude. For a single sensor at position **r**_*m*_, the time-varying electric field potential due to a single dipole of scalar amplitude *s*(*t*) is given by:

\begin{align*} \phi\left(\mathbf{r}_m,t\right) \; = \; \mathbf{g}\left(\mathbf{r}_m,\mathbf{r}_q\right)\cdot \mathbf{q}\left(t\right) \; = \; \mathbf{g}\left(\mathbf{r}_m,\mathbf{r}_q\right)\cdot \boldsymbol{U}\left(\mathbf{r}_q\right) s\left(t\right),\end{align*}

where $\mathbf{g}(\mathbf{r}_m,\mathbf{r}_q)\in\mathbb{R}^3$ is the lead-field vector per unit dipole moment, taking into account all boundaries.

Let $\{\mathbf{r}_m\ \in \mathbb{R}^3\}$ represent the set of *N_m_* sensor coordinates. We now define the forward-field vector $\mathbf{a}(\{\mathbf{r}_m\},\mathbf{r}_q) = [\mathbf{g}(\{\mathbf{r}_m\},\mathbf{r}_q)\cdot \boldsymbol{U}(\mathbf{r}_q)]$ as a vector transfer function to model the set of *N_m_* measurements generated by the single with fixed orientation $\boldsymbol{U}(\mathbf{r}_q) \in \mathbb{R}^3$. For compactness, we now suppress the dependency of $\mathbf{a(\mathbf{r}_q})$ on the set of sensor locations **r**_*m*_. The vector $\mathbf{m}(t)\in\mathbb{R}^N_m$ represents time-varying sensor data, given by (7):

\begin{align*} \mathbf{m}\left(t\right) \; = \; \mathbf{a}\left(\mathbf{r}_q\right)\,s\left(t\right) + \mathbf{n}\left(t\right),\end{align*}

where our model now includes a vector of noise components $\mathbf{n}(t)$ at the sensors, added to the model.

We now extend (7) to include the simultaneous activations of *p* dipoles, which by the principle of electromagnetic superposition, is given by

\begin{align*} \mathbf{m}\left(t\right) \; = \; \mathbf{A}\,\mathbf{s}\left(t\right) \;+\; \mathbf{n}\left(t\right),\end{align*}

where $\mathbf{A} = [\mathbf{a}(\mathbf{r}_{q1}), \ldots, \mathbf{a}(\mathbf{r}_{qp})]$ is the forward field matrix generated by *p* dipoles, and $\mathbf{s}(t)$ is the vector of corresponding dipole amplitudes.

Finally, for *T* discrete time samples, we concatenate the measurements into the matrix $\mathbf{M} = [\mathbf{m}(t_1), \ldots, \mathbf{m}(t_T)]$, and we similarly concatenate the dipole time series into $\mathbf{S} = [\mathbf{s}(t_1), \ldots, \mathbf{s}(t_T)]$, to yield the spatiotemporal model (9):

\begin{align*} \mathbf{M} \; = \; \mathbf{A}\,\mathbf{S} \;+\; \mathbf{N}.\end{align*}

### 4.2. Current-density reconstruction

Current-density reconstruction methods estimate a three-dimensional map of distributed neural activity by solving for the amplitudes of thousands of fixed dipoles, treated as image pixels. These methods produce a regularized estimate of cortical or volumetric current density that, under the assumed forward and noise models, approximately reproduces the measured data. Spatial fidelity is governed by the method’s resolution properties, including point-spread and cross-talk functions, and the ability to separate concurrent generators depends on their overlap and on the effective rank of the measurements. Within this family, minimum $\ell_2$-norm estimation (MNE) is the baseline estimator. Standardized outputs such as dynamic statistical parametric mapping (dSPM) and standardized low-resolution electromagnetic tomography (sLORETA) are variance-normalized statistics rather than physical current density and should be interpreted accordingly.

Reconstructing the amplitudes of thousands of dipoles with fixed orientations that form the ‘pixels’ of a cortical ‘image’ from only a few hundred scalp sensors creates a highly underdetermined inverse problem. This problem is similar to attempting to recover a detailed picture from a very sparse set of measurements, that is often encountered in the field of image processing. To obtain a meaningful solution, additional constraints are imposed through regularization and priors that encode assumptions about the spatial or statistical structure of neural activity.

*Bayesian formulation.* To estimate the amplitudes of dipole moments for every vertex of the cortical mesh that make up the min-norm image **S** from the data matrix **M**, the model is given by the linear equation discussed earlier (8).

Under the Bayesian framework introduced by [218], the current-density reconstruction is estimated by maximizing the log-posterior probability:

\begin{align*} \widehat{\mathbf{S}} = \underset{\mathbf{S}}{\arg\max}\; \left[ \ln p\left(\mathbf{M}\mid\mathbf{S}\right) \;+\; \ln p\left(\mathbf{S}\right) \right],\end{align*}

where $\widehat{\mathbf{s}}$ denotes the estimated dipole amplitudes for every vertex of the cortical mesh in source space; $p(\mathbf{M}\mid\mathbf{S})$ denotes the conditional probability for the data matrix **M** given the source ‘image’ **S**; finally, $p(\mathbf{S})$ denotes the prior distribution reflecting the knowledge of the statistical properties of the unknown image.

The log-likelihood is then given by

\begin{align*} \ln p\left(\mathbf{M}\mid\mathbf{S}\right) \; = \; -\frac{1}{2\sigma_\mathrm{n}^{2}} \, \bigl\| \mathbf{M}-\mathbf{A}\mathbf{S} \bigr\|_\mathrm{F}^{2}\end{align*}

where $\sigma_\mathrm{n}$ is the standard deviation of the noise, which is assumed to be temporally and spatially white.

The prior probability distribution over the cortical source matrix is modeled as an exponential family:

\begin{align*} p\left(\mathbf{S}\right) = \frac{1}{z}\exp\!\left\{-\beta\,h\left(\mathbf{S}\right)\right\},\end{align*}

where *z* is a normalizing constant, *β* is a scaling parameter, and $h(\mathbf{S})$ encapsulates the assumed statistical structure of the currents. We note that the decision to model the prior distribution as an exponential family makes the model both tractable and flexible, since it can represent many common distributions (e.g. Poisson, Bernoulli, Gaussian). However, the non-stationarity and non-normality of neural source activity may lead to deviations between the model and the observed data. The Bayesian framework for neural source estimation continues to receive attention from researchers to address its limitations, as newer approaches are discussed in section 4.3 and section 4.4.

#### 4.2.1. MNE

MNE represents the standard baseline solution derived from this Bayesian formulation, specifically when a Gaussian distribution is selected for the prior.

Combining the log-likelihood (11) with the log-prior (12) produces the negative log-posterior; minimizing this quantity yields the maximum *a posteriori* (MAP) estimate, which corresponds to a weighted minimum $\ell_2$-norm solution. As the model is assumed to be prewhitened [24], the log prior in (12) has the form

\begin{align*} h\left(\mathbf{S}\right) \; = \; \operatorname{tr}\!\left\{ \mathbf{S}\, \mathbf{C}_\mathrm{s}^{-1}\, \mathbf{S}^{\mathsf{T}} \right\},\end{align*}

where, $\mathbf{C}_\mathrm{s}$ is the spatial covariance matrix of the min-norm image.

Minimizing the negative log-posterior in (10) with the Gaussian prior yields the MAP estimator

\begin{align*} \widehat{\mathbf{S}}_\mathrm{MNE} \; = \; \mathbf{C}_\mathrm{s}\mathbf{A}^{\mathsf{T}} \left( \mathbf{A}\mathbf{C}_\mathrm{s}\mathbf{A}^{\mathsf{T}} + \mathbf{C}_\mathrm{n} \right)^{-1} \mathbf{M} \;\equiv\; \mathbf{F}\,\mathbf{M}.\end{align*}

where **F** denotes the linear inverse operator formed by the MAP estimator.

When the noise is assumed white such that $\mathbf{C}_\mathrm{n} = \lambda\mathbf{I}$, this reduces to the Tikhonov-regularized MNE, with *λ* the regularization parameter. In signal processing, this estimator is equivalently known as the Wiener estimate or linear minimum mean-squared error solution.

#### 4.2.2. dSPM

dSPM [213] involves normalization of the MNE, estimated using (14), by its estimated noise variance at each location, similar to neural activity index (NAI) (discussed in section 4.6). In Bayesian terms, dSPM does not assume a new prior but rather computes a statistical *Z*-score map of the white Gaussian posterior, which represents an estimate of SNR. This normalization is accomplished by estimating the noise sensitivity $\Sigma_{\widehat{\mathbf{s}}}$ :

\begin{align*} \boldsymbol{\Sigma}_{\widehat{\mathbf{s}}} \; = \; \mathbf{F}\,\mathbf{C}_\mathrm{n}\,\mathbf{F}^\mathsf{T}.\end{align*}

The noise-normalized *Z*-score estimate (**z**_*i*_) at source location *i*, is computed by normalizing the amplitude $\widehat{\mathbf{s}}$ with the estimate of noise sensitivity:

\begin{align*} \mathbf{z}_i\left(t\right) \; = \; \frac{\widehat{\mathbf{s}}_i\left(t\right)}{\sqrt{\,\left[\boldsymbol{\Sigma}_{\widehat{\mathbf{s}}}\right]_{ii}\,}}.\end{align*}

This has three effects: (i) it transforms the map into dimensionless statistical scores, (ii) it reduces the well-known superficial-depth bias of MNE, and (iii) it makes the point-spread function more uniform across the cortex.

#### 4.2.3. LORETA/sLORETA family

LORETA [219] imposes spatial smoothness via a zero-mean Gaussian prior on the source distribution whose precision is proportional to a discrete Laplacian smoothness operator defined on the source grid or cortical mesh.

Let **$\mathbf{D}$** denote the Laplacian operator and $\mathbf{R} = \mathbf{D}^{\mathsf{T}}\mathbf{D}$ the smoothness precision. In (14), LORETA derives the source covariance using the smoothness precision as $ \mathbf{C}_\mathrm{s} \;\propto\; \bigl(\mathbf{R}+\lambda\mathbf{I}\bigr)^{-1} $, which penalizes spatial roughness of the estimate. sLORETA [220] uses essentially the same prior as in (12). Moreover, like dSPM, it is a noise-normalized form of MNE that yields unitless, variance-standardized scores at each location; it is not a current-density distribution. The single-source unbiased localization result holds under correct forward and noise models; for multiple simultaneous generators the image reflects linear superposition of point-spread functions and source separation is not guaranteed [220, 221].

In Bayesian terms, all sLORETA variants use Gaussian priors but with nontrivial covariance. sLORETA can also be viewed as MAP [222]:

\begin{equation*} \widehat{\mathbf{S}}_\textrm{sLORETA} = \mathbf{A}^{\mathsf{T}}\left(\mathbf{A}\mathbf{A}^{\mathsf{T}} + \lambda^2 \mathbf{I}\right)^{-1}\mathbf{S}.\end{equation*}

It further scales each solution component by the inverse square root of its variance, as in dSPM [213]. Under idealized single-source conditions with correct forward and noise models, sLORETA achieves zero localization bias [220]. In noisy data, whether sLORETA remains unbiased depends on the assumed noise model and SNR [223]. Recent analysis further shows that sLORETA is exactly equivalent to single-dipole scanning in an appropriate inner-product space, which clarifies why the unbiasedness guarantee is limited to single-source scenarios [224].

Exact LORETA (eLORETA) [225] goes further by analytically constructing a weight matrix that achieves ‘exact, zero-error’ localization even in the presence of measurement noise. These methods tend to produce smoother, more distributed maps than MNE, at the expense of depth bias in plain LORETA. However, sLORETA/eLORETA mathematically correct the bias [47, 225].

### 4.3. Hierarchical Bayesian frameworks

Hierarchical Bayesian models introduce multiple layers of prior distributions, allowing for adaptive regularization and the incorporation of anatomical, spatial, and statistical constraints. These models can capture both focal and distributed sources, as well as multiscale spatial features. For example, randomized multiresolution scanning (RAMUS) [226] leverages a hierarchical Bayesian approach to achieve robust and accurate source localization across both superficial and deep brain regions, without requiring *a priori* knowledge of the number or location of active sources. RAMUS uses an inverse-gamma hyperprior and randomized scanning to reduce discretization and optimization errors, enhancing the visibility of deep sources and providing robust MAP estimates for primary current density [227]. This approach has been reported to outperform in scenarios involving simultaneous thalamic and somatosensory activity. Another hierarchical approach used Markov Chain Monte-Carlo techniques to efficiently compute the MAP estimates [228]. More recent techniques introduced explicit structural constraints: one approach leveraged hierarchical graph priors via spanning trees to effectively handle noise while maintaining spatiotemporal continuity among neural sources [229], while the *µ*-STAR model incorporated microstate detection to inform the hierarchical priors [230]. Beyond these methods, other hierarchical models, such as those employing structured sparsity priors, variational sparsity, or multiscale graphical models, further enhance the resolution of sources with varying spatial extent and facilitate the separation of closely spaced or correlated sources [231–233]. These advances are particularly valuable for clinical applications, such as epilepsy localization, where both accurate depth localization and source separation are critical.

### 4.4. Empirical Bayesian approaches

Empirical Bayesian methods estimate hyperparameters directly from the observed data, rather than fixing them *a priori*. This is particularly powerful for modeling noise, which in real EEG/MEG recordings is often structured and non-Gaussian. The structured noise Champagne algorithm exemplifies this approach by jointly estimating brain source activity and structured noise statistics using a variational Bayesian factor analysis model [234, 235]. Unlike traditional methods that assume white or stationary noise, this framework can adapt to spatially correlated environmental and biological noise sources, leading to more accurate and robust source reconstructions. Notably, it does not require separate baseline measurements, making it suitable for scenarios where noise characteristics change dynamically or are only present during active periods.

Recent work has also extended empirical Bayesian frameworks to handle full noise-covariance structure estimation, further improving robustness in real-world scenarios where noise is non-diagonal and highly structured [236, 237]. These methods are particularly effective in low SNR conditions and for distributed source configurations.

### 4.5. ECD reconstruction

ECD reconstruction posits that the measurements arise from a small number of focal generators. Each generator is represented as an ECD with unknown location and time-varying amplitude, yielding a discrete solution once the parameters are identified. For clarity, we review two canonical families among many available ECD reconstruction methods: least-squares estimation, which performs a direct non-linear search for the optimal dipole parameters that minimize the residual error, and Subspace scanning methods, such as multiple signal classification (MUSIC) and its recursive variant recursively applied and projected MUSIC (RAP-MUSIC). These methods use the sensor covariance structure to separate signal and noise subspaces and then test candidate cortical locations for consistency with the estimated signal subspace [46, 238]. RAP-MUSIC iterates this test with deflation to localize multiple generators without an explicit nonlinear fit of amplitudes.

When the sparsity assumption holds and SNR is adequate, ECD reconstruction methods provide high spatial specificity and interpretable, discrete solutions that complement distributed minimum-norm imaging [24].

#### 4.5.1. Least-squares estimation

Least-squares source estimation represents one of the earliest and most straightforward strategies for solving the inverse problem. Building on the spatiotemporal model defined in (9), the goal is to determine the set of dipole parameters that best describe the data matrix **M** in the presence of measurement noise. In this framework, the forward field matrix **A** depends nonlinearly on the dipole locations $\{\mathbf{r}_{qi}\}$, while the dipole amplitude time series **S** represents the linear parameters. The estimation seeks to minimize the squared error between the measured data and the fields predicted by the forward model. The measure of fit is defined as the square of the Frobenius norm:

\begin{align*} \mathcal{J}_{LS}\left(\{\mathbf{r}_{qi}\}, \mathbf{S}\right) \; = \; \|\mathbf{M} - \mathbf{A}\left(\{\mathbf{r}_{qi}\right)\mathbf{S}\|_\mathrm{F}^{2}.\end{align*}

While a simultaneous nonlinear search over all parameters is possible, it is computationally burdensome. However, for any fixed set of locations and orientations, the amplitude matrix **S** that minimizes (17) can be determined analytically as $\widehat{\mathbf{S}} = \mathbf{A}^{+}\mathbf{M}$, where $\mathbf{A}^{+}$ is the pseudoinverse of **A**. Substituting this optimal amplitude back into the cost function allows the problem to be separated, requiring minimization solely over the nonlinear parameters:

\begin{align*} \mathcal{J}_{LS}\left(\left\{\mathbf{r}_{qi}\right\}\right) \; = \; \|\mathbf{M} - \mathbf{A}\mathbf{A}^{+}\mathbf{M}\|_\mathrm{F}^{2} \; = \; \|\boldsymbol{\Pi}_{\mathbf{A}}^{\perp}\mathbf{M}\|_\mathrm{F}^{2}\end{align*}

where $\boldsymbol{\Pi}_{\mathbf{A}}^{\perp} = \mathbf{I} - \mathbf{A}\mathbf{A}^{+}$ is the orthogonal projection matrix onto the left null space of **A**. The nonlinear parameters are then estimated using iterative minimization algorithms such as Levenberg–Marquardt or Nelder–Mead simplex searches. This model can be applied sequentially to individual time slices to form a ‘moving dipole’ model, or to the entire data block to constrain the location as fixed over the interval.

However, the least-squares approach faces a critical computational bottleneck when modeling multiple sources. As the number of sources increases, the dimension of the parameter space grows, and the cost function becomes highly non-convex, resulting in a landscape riddled with local minima. Consequently, simultaneous non-linear searches for multiple dipoles are prone to trapping and depend heavily on the accuracy of the initial guess. This limitation motivates the use of scanning approaches that can localize multiple sources without performing a high-dimensional non-linear optimization.

#### 4.5.2. Subspace scanning methods

Subspace scanning methods, originally developed in array processing for multi-source direction finding, were adapted to EEG/MEG to decouple location testing from amplitude fitting. Exemplified by MUSIC, as applied by Mosher *et al* [238], these techniques exploit the sensor covariance structure to partition signal and noise subspaces, enabling the scanning of candidate locations without explicit nonlinear amplitude estimation.

For the model in (9), the data matrix **M** can be decomposed using singular value decomposition to form $\mathbf{{\mathbf{M}}} = \boldsymbol{U}\boldsymbol{\Sigma} \mathbf{V}^\mathsf{T}$. Assuming the independent and identically distributed (IID) noise, the set of left singular vectors (**U**) can be partitioned into signal and noise-only subspace. The signal subspace is spanned by the first *p* singular values (denoted by **U**_*s*_), and the noise-only subspace is spanned by the remaining left singular vectors. As a result, the best rank-*p* approximation of $\mathbf{m}(t)$ is obtained as $\mathbf{m}_s(t) = (\boldsymbol{U}_s \boldsymbol{U}_s^\mathsf{T})\cdot\mathbf{m}(t)$, with the corresponding noise-subspace projection operator defined as $\boldsymbol{\Pi}^\perp_s = \mathbf{I} - (\boldsymbol{U}_s \boldsymbol{U}_s^\mathsf{T})$. The MUSIC localizer is then expressed in terms of this operator, yielding the cost function [238]:

\begin{align*} \mathcal{J}\left(\mathbf{r}_{qi}\right) &amp; = \frac{\| \boldsymbol{\Pi}^\perp_s \mathbf{a}\left(\mathbf{r}_{qi}\right) \|^2_2}{\|\mathbf{a}\left(\mathbf{r}_{qi}\right)\|_2^2}\end{align*}

where $\mathcal{J}(\mathbf{r}_{qi})$ denotes the MUSIC cost function evaluated at dipole location *q* at time *i*, and $\boldsymbol{\Pi}^\perp_s$ denotes the perpendicular projection operator into noise-only subspace.

MUSIC cost function is zero when $\mathbf{a}(\mathbf{r}_{qi})$ corresponds to the true locations of the sources. Therefore, the inverse solution is found by searching for maxima in the ‘MUSIC’ scan, defined as the reciprocal of the cost function: $P_{\textrm{MUSIC}} = 1/\mathcal{J}(\mathbf{r}_{qi})$ where peaks indicate the estimated location of the dipoles. Intuitively, MUSIC finds locations whose predicted field patterns correlate strongly with the measured data’s dominant components. One advantage is that it does not require inverting the full covariance and can handle temporally correlated sources better than the beamformer (discussed in section 4.6) algorithms, since uncorrelated source assumptions are not needed in its localizer.

RAP-MUSIC [46] uses MUSIC recursively to localize multiple simultaneous sources. RAP-MUSIC works iteratively by finding the best location for one source via MUSIC, then projecting the data into the subspace orthogonal to that source’s field, and therefore effectively ‘removing’ its contribution, and then finds the next source location in the residual data. This recursive projection continues until the desired number of sources is found. RAP-MUSIC thereby automates multi-dipole localization, addressing the peak-finding ambiguity of basic MUSIC and allowing a straightforward multi-source search. Notably, RAP-MUSIC inherits key assumptions of MUSIC: it presupposes predominantly uncorrelated, focal sources and requires the number of sources (or a stopping threshold) to be set. This could be mitigated by employing metrics such as Akaike information criteria [239], Bayesian information criteria [240], minimum description length [239], and F-ratio [241].

Over the years, several variants of RAP-MUSIC have been introduced, which extend the functionality of RAP-MUSIC. Truncated RAP-MUSIC (TRAP-MUSIC) [242] was proposed to improve robustness in estimating the number of sources. RAP-MUSIC can leave behind residual variance that distorts subsequent iterations. TRAP-MUSIC addressed this issue by performing a sequential reduction of the signal subspace dimension at each iteration. This is achieved by truncating the remaining eigenspectrum by one (or the estimated multiplicity) of that source’s eigenvalues, thereby ensuring that residual unexplained variance is pushed into the noise subspace. FLEX-MUSIC [243] and its sibling FLEX-AP [244] push the MUSIC family beyond the point-dipole assumption by letting each candidate generator adopt a variable spatial extent. They precompute a dictionary of leadfield ‘atoms’ ranging from single dipoles to smoothly smoothed cortical patches; during scanning, the algorithm can pick whichever extent best fits the residual data. FLEX-MUSIC [243] keeps RAP’s eigenprojection framework, whereas FLEX-AP [244] embeds this idea in the alternating-projection (AP) solver that is inherently more tolerant of temporally correlated sources. Moving to the frequency domain further broadens MUSIC’s reach. Self-consistent MUSIC works on the imaginary part of the cross-spectral density (CSD), which was created to help localize a specific brain rhythm, such as *µ* rhythms (∼11 Hz) [245].

### 4.6. Spatial filtering

Spatial filtering refers to the design of beamformer filters that allow the passage of activity from a designated location while attenuating signals from elsewhere, thereby providing spatially selective sensitivity. Intuitively, the LCMV beamformer asks whether one can construct a filter that ‘listens’ to a single location while suppressing all others. Mathematically, beamforming seek the weight matrix $\mathbf{W}\in\mathbb{R}^{N_m \times p}$ that transforms the data $\mathbf{m}(t)$ to source activity $\mathbf{s}(t)$. Originally developed for radar and sonar applications, beamforming has become a widely used approach for source localization in MEG and EEG.

The seminal paper by Van Veen *et al* [214, 246, 247] introduced the LCMV beamformer algorithm, which applied Capon/minimum variance distortionless response [248] beamformer theory to localize brain electrical activity. The algorithm designs a weight vector $\mathbf{W^{\mathsf{T}}}$ for each candidate source location **r**_*q*_ such that (i) the beamformer output ($\mathbf{s}(t) = \mathbf{W}^{\mathsf{T}}\mathbf{m}(t)$) has unit gain for a dipole at that location, and (ii) the output power of sources originating from other locations is minimized. Assuming fixed orientation dipoles and the model in (7), the mathematical formulation of estimating $\mathbf{W^{\mathsf{T}}}$ is given as:

\begin{align*} \arg\min_{\mathbf{W}^{\mathsf{T}}} \operatorname{tr}\!\left\{\mathbf{C}_\mathrm{s}\right\} \quad\mathrm{s.t.}\quad \mathbf{W}^{\mathsf{T}}\mathbf{a}\left(\mathbf{r}_q\right) = \mathbf{I},\end{align*}

where $\mathbf{C}_\mathrm{s} = \mathbb{E}\!\bigl[\mathbf{s}\mathbf{s}^{\mathsf{T}}\bigr] = \mathbf{W}^{\mathsf{T}}\mathbf{C}_m\mathbf{W}$ denotes the output covariance matrix. The data covariance $\mathbf{C_m}$ is estimated empirically via $\mathbb{E}\!\bigl[\mathbf{m}\,\mathbf{m}^{\mathsf{T}}\bigr] = \mathbf{M}\mathbf{M}^\mathsf{T}/T$.

Solving (20) using the method of Lagrange multipliers leads to the following solution [246]:

\begin{align*} \mathbf{W}^\mathsf{T} = \left[ \mathbf{a}^\mathsf{T}\left(\mathbf{r}_q\right) \, \mathbf{C}_m^{-1} \, \mathbf{a}\left(\mathbf{r}_q\right) \right]^{-1} \mathbf{a}^\mathsf{T}\left(\mathbf{r}_q\right) \, \mathbf{C}_m^{-1}.\end{align*}

To compensate for depth bias and noise, Van Veen *et al* proposed the NAI [214], Intuitively, NAI is akin to a SNR metric highlighting locations with activity above the noise floor, where the peaks correspond to the putative source locations. It is computed by normalizing the output variance of the LCMV beamformer, computed using (21), with the output obtained in the presence of noise only:

\begin{align*} \mathrm{NAI}\left(\mathbf{r}_q\right)\; = \; \frac{\operatorname{tr}\!\left\{\left[\mathbf{a}^{\mathsf{T}}\left(\mathbf{r}_q\right)\,\mathbf{C}_{m}^{-1}\,\mathbf{a}\left(\mathbf{r}_q\right)\right]^{-1}\right\}} {\operatorname{tr}\left\{\!\left[\mathbf{a}^{\mathsf{T}}\left(\mathbf{r}_q\right)\,\mathbf{C}_{n}^{-1}\,\mathbf{a}\left(\mathbf{r}_q\right)\right]^{-1}\right\}}\end{align*}

where, $\mathbf{C}_\mathrm{n} = \mathbb{E}\!\bigl[\mathbf{n}\,\mathbf{n}^{\mathsf{T}}\bigr]$ is the noise covariance.

An advantage of the LCMV beamformer is that it makes no assumption on the number of active sources, instead exploiting the full sensor covariance; it can thus image multiple sources without specifying how many are present. Moreover, the beamformer algorithms need a relatively few number of user-specified parameters, namely, the size of the reconstructed grid, the time-frequency window of interest, and noise regularization parameters [249].

Over the last 25 years, numerous extensions to the basic LCMV beamformer approach have been introduced to address limitations of classic LCMV beamforming methods [250–255]. A well-known limitation is their sensitivity to partially correlated sources; if two brain regions are synchronous, a beamformer may erroneously suppress both, as the covariance matrix does not offer a unique solution for their separate contributions. Various strategies have been devised to modify the beamformer constraint to allow the passage of multiple simultaneous sources. One landmark example is the dual-source beamformer proposed by Brookes *et al* [256], which was originally devised for MEG, but later applied to EEG [257]. In this approach, the leadfield is re-formulated to allow two spatially distinct target locations instead of one. Essentially, two weight vectors are computed as a coupled system, allowing a pair of highly correlated sources to be imaged without canceling each other. Another approach, which is known as the nulling beamformer (NB), was introduced by Hui *et al* [258]. The NB approach imposes additional linear constraints to explicitly suppress activity from other known regions while passing the target region.

Another limitation is the algorithm’s high degree of sensitivity to the forward modeling errors. EEG forward models are sensitive to head geometry and tissue conductivities; errors in electrode co-registration or misspecification of skull conductivity can lead to a mismatch between the assumed leadfield and the true leadfield. To reduce the sensitivity of beamformers to the errors in forward modeling, the so-called model mismatch problem, robust beamformers were introduced. Robust minimum variance beamformer introduced by Hosseini *et al* [259] empirically estimated uncertainty ellipsoids for each location by sampling neighboring points and different head models, then solved for robust weights. Additionally, because the beamformer must invert the covariance matrix, an ill-conditioned or poorly estimated covariance can introduce instability and require regularization. To deal with this problem, several robust covariance estimation techniques such as probabilistic principal component analysis [260], the minimum-covariance-determinant estimator [261], and Ledoit–Wolf linear shrinkage [262] were introduced.

Finally, LCMV beamformers also suffer from a depth bias; sources deeper in the brain have lead fields with smaller norms, resulting in larger weight norms and artificially inflated power estimates for superficial sources. In order to address these problems, spatial normalization strategies were employed, which involve leadfield normalization at the filter weight computation step [263]; examples include array-gain beamforming [255] and unit-noise-gain beamforming [264].

Another specialized beamformer approach called dynamic imaging of coherent sources (DICSs), introduced by Gross *et al* [265], extends the beamforming approach to the frequency domain. Unlike a time-domain beamformer, which maximizes output power, DICS constructs spatial filters to localize regions that are coherently oscillating with a given reference or with each other and allows functional connectivity estimations. The algorithm computes the CSD matrix of sensor signals at the frequency of interest (e.g. an epileptic oscillation at 5 Hz or a beta rhythm at 20 Hz). DICS then designs a spatial filter $\mathbf{W}(\mathbf{r}_q)$ for each location such that it passes signals from **r**_*q*_ and simultaneously maximizes the coherence between the output at **r**_*q*_ and a target signal.

### 4.7. Other source reconstruction approaches

Beyond ECD reconstruction, current-density reconstruction, and spatial filtering methods, several complementary families of source reconstruction methods operate on the same linear forward model but impose different structural assumptions or optimization criteria. These approaches are often chosen to match a specific scientific or clinical aim, for example, detecting spatially extended generators, or separating mixed processes.

Entropy-based distributed methods, typified by the maximum entropy on the mean framework, replace quadratic smoothness with information-theoretic priors and parcel-level organization, yielding solutions that can adapt to both focal and extended generators while remaining data-driven. Blind source separation (BSS) techniques, especially independent component analysis (ICA) coupled with dipole fitting, treat the problem as statistical demixing; they are widely used to isolate physiologically plausible, often dipolar, components and to remove artifacts, thereby simplifying subsequent localization. Metaheuristic global optimization methods such as particle swarm optimization (PSO) recast localization as a direct search in the nonlinear parameter space of ECDs; by relying only on the forward operator, they can escape local minima and handle model orders that are difficult for gradient-based fits, at higher computational cost.

In the subsections that follow, we discuss other techniques, emphasizing when they are advantageous, their principal assumptions, and common pitfalls relative to the methods established in the previous sections.

#### 4.7.1. Maximum entropy of mean (MEM)

MEM is a Bayesian distributed source imaging framework that incorporates minimal prior assumptions by maximizing an entropy-like measure of uncertainty subject to the data constraints. In practice, the source amplitudes **s** are modeled as random variables. MEM finds the source distribution that is maximally non-committal (maximum entropy) except as required to fit the observed scalp data **m**, via the model is given by (7).

Conceptually, one posits a probability density function $P(\mathbf{s})\propto \exp(-\Phi(\mathbf{s}))$ and chooses $\Phi(\mathbf{s})$ to maximize entropy subject to data fidelity. Clarke and Janday [266] first applied maximum-entropy ideas to the biomagnetic inverse problem, and Rice [267] discussed the neurophysiological justification of maximum-entropy EEG solutions

The critical prior in modern MEM methods is a data-driven cortical parcellation. Brain sources are assumed to be organized in non-overlapping spatial parcels of the cortex, each of which may be ‘active’ or not. Within each active parcel, sources are allowed a contrast of intensity. Early MEM implementations used anatomically-informed parcels, but later approaches use data-driven parallelization. In this model, the unknown source vector **s** is partitioned into blocks corresponding to parcels; MEM infers which parcels are active and with what intensity. This yields sensitivity to spatially extended generators as well as focal ones. Grova *et al* [268] showed that MEM can recover both the location and extent of simulated epileptic spikes. Chowdhury *et al* [269] confirmed that MEM methods detect sources from very small (∼3 ${\mathrm{cm}^2}$) up to large extents (∼30 $\mathrm{cm}^2$) with high accuracy.

Coherent MEM (cMEM) [269] applies to time-domain evoked or averaged data and assumes the cortical parcels are temporally stable over the analysis window. It computes a single inverse solution (source map) that best fits the data while respecting parcel-level smoothness. Wavelet MEM (wMEM) [270] extends MEM into the time–frequency domain: the EEG signals are decomposed into discrete wavelet bands, and MEM is applied separately to each band. This is suited for localizing oscillatory activity (e.g. gamma bursts) at specific times, using a parcellation that can vary with frequency. Ridge MEM (rMEM) [271] targets synchrony patterns: it uses a continuous complex-wavelet transform to detect intervals of high phase-locking across channels and localizes the synchronous generators of those patterns. In summary, cMEM recovers static/evoked sources, wMEM localizes oscillatory bursts, and rMEM isolates phase-coherent activity.

#### 4.7.2. ICA/DipFit

The source reconstruction problem is also formulated as a BSS problem, and ICA has been used to isolate the putative source processes. Delorme *et al* [272] provided compelling empirical evidence that maximally independent EEG components are predominantly dipolar, validating ICA as a biologically meaningful preprocessing step for EEG/MEG source imaging. Using a four-shell spherical head model, they tested 22 linear decomposition algorithms to localize sources and concluded that ICA implementations such as adaptive mixture ICA and Extended-Infomax reduced a high-dimensional distributed inverse problem to a series of low-parameter dipole fits.

#### 4.7.3. PSO (SWARM)

PSO [273] is a stochastic global optimization technique inspired by the social behavior of animals. In EEG source localization, it treats the inverse problem as a nonlinear search for dipole parameters. Minimizing **a** over **r**_*q*_ is nonlinear and nonconvex due to the inverse operation. PSO solves this by simulating a swarm of candidate solutions (‘particles’) moving through the parameter space. A ‘modified PSO’ (MPSO) extended the approach to multi-dipole scenarios [274]. The key insight is that PSO requires only interacting with the forward operator and can escape local minima better than greedy methods. PSO’s strength is its global search capability. It can find sources that gradient methods might miss and requires no initial guess.

For somatosensory-evoked potentials (SEPs) and epileptic spike localization, it provides an easy way to localize the peak generator without linearizing assumptions. Shirvany *et al* [275] applied MPSO to hd-EEG of SEP. They generated a realistic 1 mm FEM head model and restricted sources to the gray matter. They reported that MPSO converged to the true source region and dramatically outperformed exhaustive grid search (3700× fewer evaluations) in computation. The main hyperparameters are swarm size, inertia, cognitive/social factors, and maximum iterations. Shirvany *et al* used adaptive swarm sizing and special rules to avoid stalling

Unlike ICA or MEM, PSO can, in principle, handle multiple dipoles by extending the search space. However, PSO is computationally demanding: each particle update requires a forward solve and as many solves per iteration as particles. Even with 30 particles and 100 iterations, one does 3000 forward solves, which can be slow. Shirvany *et al* [273] reported PSO took hundreds of seconds for a single-source localization, versus hours for exhaustive search. PSO also needs careful tuning; poor parameter choices can cause premature convergence. In practice, PSO has a risk of trapping in local minima if the swarm diversity collapses.

### 4.8. Review of supervised learning based techniques

Supervised learning based approaches focus on utilizing supervised machine learning, especially deep learning, to directly learn a nonlinear function between scalp EEG and underlying brain source activity and location. These methods do not typically employ a fully data-driven approach, instead opting to use advanced models of brain activity to simulate scalp EEG and source activity for model training. Deep learning models benefit from enhanced representation learning that can capture more complex relationships in data that are often present in the field of brain source localization. Due to the nature of deep learning models as universal approximators, these approaches aim to find an estimate of the sources $\widehat{\mathbf{S}}$ by approximating the inverse operator $\widehat{f}$ as in (23):

\begin{equation*} \widehat{\mathbf{S}} = \widehat{f}\left(\mathbf{M}\right).\end{equation*}

The application of deep learning methods to solve the EEG inverse problem is relatively new in the literature. Cui *et al* [276] used a long-short term memory (LSTM) recurrent neural network (RNN) to reconstruct the position and time course of one source. This work trained and tested the model on simulated data with varying degrees of noise perturbations.

Hecker *et al* [277] created a model called ConvDIP that uses a shallow convolutional neural network (CNN) model capable of solving the inverse problem using a distributed dipole solution (between 1 and 5 source clusters). The model was trained using synthetic data generated from a biophysical patch–source model and was then tested on real scalp EEG recordings from a single subject.

Sun *et al* [45] utilized a deep learning framework called DeepSIF that combined CNN and RNN architectures to leverage both spatial (position of electrodes from scalp EEG) and temporal (model the temporal dynamics of brain sources) information in scalp EEG data. Additionally, this work employed NMMs, which are mathematical models used to simulate the average activity of neuronal populations in the brain. They simplify the complex dynamics of individual neurons by representing them as interconnected masses of neurons, focusing on the overall firing rates and membrane potentials of these populations. This work segmented the brain into 994 regions of possible source activity, greatly improving the spatial-temporal resolution of DNN-based solutions for solving the EEG inverse problem. The model trained on the synthetic data was then tested on three publicly available scalp EEG datasets and one epilepsy dataset to validate the model.

Wu *et al* [278] developed a deep learning framework based on manifold learning to address the EEG inverse problem. This methodology utilizes a variational autoencoder that first learns how to compress and subsequently reconstruct scalp EEG activity in an unsupervised manner. The goal of an autoencoder is to learn a low dimensional latent space in which most of the variability in the neural data exists and can be modeled as a nonlinear manifold. Once this latent space is learned, scalp measurements can be projected into the latent space and input into a decoder module that learns how to reconstruct intracranial measurements. This model was trained on synthetic data using 226 regions of source activity and then tested on two public datasets of scalp EEG focused on epileptic activity and visual evoked potentials.

### 4.9. Direct mapping of scalp EEG to iEEG activity

Previous methods to solve the EEG inverse problem rely heavily on a robust model of brain dynamics to generate synthetic scalp EEG and source activity for model training. These methods then test their models on additional synthetic data, which can greatly inflate the accuracy in the model reconstructions. In addition, these methods can test the models on public scalp EEG datasets to check that the model estimates source activity in the generally correct area, such as seizure foci or the visual cortex for visual evoked response experiments. However, these methods do not have a true ground truth measurement of electrical activity within the brain to evaluate these methods.

An alternative approach to address the inverse problem would be to obtain synchronized scalp and iEEG and then train a model to reconstruct the intracranial measurements from the scalp measurements. Obtaining a dataset of this nature is difficult as iEEG would require an invasive procedure, proper labeling of neural sources, and a wide distribution of sources measured. However, drug-resistant epilepsy patients routinely undergo a sEEG procedure, which involves the placement of 10–20 thin wire electrodes through small holes in the skull to record brain activity and identify the epileptogenic zone. Scalp EEG is often acquired simultaneously to aid in the localization of seizure activity. Patients are implanted for several days to a few weeks and are tapered down on anti-seizure medications in order to monitor bona fide epileptic activity. Data acquired from this type of procedure is perfectly suited for a fully data-driven approach to solving the inverse EEG problem, which could potentially remove the need for complex modeling of brain activity entirely or be used in tandem with neural models to aid in inverse model development. While synchronous recordings of sEEG and scalp EEG may originate in epilepsy monitoring units, the applications for this technique would extend to numerous applications, such as those discussed in section 5.

The first work to directly map scalp EEG to sEEG data using DNN’s was Antoniades *et al* [279]. This work utilized an asymmetric autoencoder to map the temporal sequence of scalp EEG to sEEG. The converted signals were then fed into a CNN architecture to classify if the epoch of data contained an intracranial epileptic discharge (IED). This method outperformed all previously developed linear methods. Took *et al* [280] used a similar approach with autoencoders, but used pretrained models to improve the model training and classification accuracy. Hu *et al* [281] expanded this work by using a generative adversarial network (GAN) to generate sEEG signals from scalp EEG. GAN’s consist of two steps. First, an encoder-decoder model learns how to generate sEEG from scalp EEG data using classic DNN architectures like CNN’s or RNN’s. Second, a discriminator model aims to correctly classify between real examples of sEEG data and the synthesized data. Both models are trained simultaneously in a competitive manner such that the first model generates higher fidelity synthetic sEEG and the second model learns how to expertly discern between real and synthetic data. In this specific work, they modified the discriminator to compare the time series representations, frequency spectra, and spatial correlations between EEG channels to produce a high fidelity mapping. This work did not apply the method to IED classification, limiting the applicability. Abdi *et al* [282] used a combination of autoencoders and GANs to directly map scalp EEG to sEEG and for the eventual prediction of IED periods.

To the authors’ best knowledge, there is currently no methodology that combines synthetic data and real data for the training of DNN architectures to solve the EEG inverse problem. Future research combining these approaches may lead to superior performance and more generalizable models to unseen patients, expanding the clinical utility of these methods.

### 4.10. Inverse model conclusions

This section synthesized key techniques for EEG source analysis. The inverse problem, which is notoriously ill-posed, seeks to reconstruct the underlying brain sources and has traditionally relied on either ECD reconstruction for focal sources or minimum-norm imaging for distributed activity, both of which use regularization to find a plausible solution. Among ECD reconstruction approaches, MUSIC demonstrates more stability with respect to correlated sources compared to beamforming approaches, while among current-density reconstruction approaches, the choice among formulations depends on the assumed covariance of the data and desired depth of imaging. We summarize these key linear approaches and their assumptions, use cases, and limitations in table 2. Recent advancements, including the direct mapping of scalp EEG to intracranial recordings and supervised machine learning techniques (summarized in table 3), offer new avenues for solving the inverse problem by bypassing complex biophysical models and leveraging ground-truth data from patients. Future directions aim to leverage advances in intracranial measurements, computational hardware, and multimodal, data-driven frameworks for improved accuracy, generalizability, and downstream clinical utility.

**Table 2.** Comparison of quasi-linear source estimation methods. This table summarizes the specific underlying assumptions, optimal application scenarios (best-use cases), and inherent limitations for a taxonomy of linear and scanning inverse algorithms.

Method	Assumptions	Best-use cases	Limitations	Reference
MUSIC/RAP-MUSIC	• Focal source • Linearly independent sources • Accurate forward model • Gaussian noise with known covariance	• Multi-dipole scans without non-linear fitting	• Sensitive to forward model error • Residual variance left behind during recursive projection • Requires subspace rank (model order) selection	[238]

LCMV beamformer	• Focal sources • Uncorrelated sources • Accurate forward model • Well-estimated data covariance • Noise wide-sense stationary • Sufficient samples per parameter	• Scanning brain-wide activity to find specific sources • Event-related desynchronization/synchronization • high-specificity functional connectivity analyses that are less contaminated by field spread.	• Break down with correlated sources or closely spaced sources • Sensitive to forward model error • Suffers from depth bias	[246]

dSPM/sLORETA	• Accurate forward model • Gaussian noise	• Imaging broad or unknown source configurations in task-based studies • Produces interpretable map of brain activity with minimal assumptions on source count • Works well with averaged data	• Output is a variance-normalized statistic • Unbiasedness holds only for a single point source • Limited spatial precision and dependency on noise estimation • Overestimate the spatial extent	[213, 220]

cMEM	• Parcel-level spatial coherence • Temporal stability within the analysis window • Imposes a Bayesian prior that maximizes entropy	• Localizing spatially extended cortical generators • Multiple simultaneous sources	• Computationally intensive and model-dependent • Lower performance for a single very focal, high-SNR source • Sensitive to parcellation and hyperparameters	[268]

ICA/DipFit	• Linear, instantaneous mixing of statistically independent source processes • Number of recoverable ICs $\unicode{x2A7D}$ number of sensors • Wide-sense stationarity over the analysis window	• Number of recoverable ICs $\unicode{x2A7D}$ number of sensors; approximate stationarity over the analysis window • Decomposing ERPs into physiologically plausible subcomponents and fitting dipoles to dipolar ICs	• Fails if the source independence assumption is violated • User intervention involved in selecting artifactual sources • Cannot separate sources that overlap in time perfectly	[272]

SWARM/PSO	• Low-dimensional global optimization problem exists	• Global search or a few dipole sources • Useful when little prior information is available • Windowed localization at event peaks or brief epochs	• Effectiveness drops with multiple or correlated sources • Computationally expensive • Convergence is not guaranteed • Hyperparameter sensitivity (swarm size, inertia, learning rates)	[273]

*Abbreviations:* **MUSIC** = multiple signal classification; **RAP-MUSIC** = recursively applied and projected MUSIC; **LCMV** = linearly constrained minimum variance; **dSPM** = dynamic statistical parametric mapping; **sLORETA** = standardized low-resolution brain electromagnetic tomography; **cMEM** = cortical maximum entropy on the mean; **ICA** = independent component analysis; **IC** = independent component; **ERP** = event-related potential; **PSO** = particle swarm optimization; **SNR** = signal-to-noise ratio.

**Table 3.** Recent supervised deep learning approaches for EEG Source estimation. This table contrasts various supervised neural network architectures applied to the inverse problem. It details the synthetic data generation strategies (utilizing forward models such as BEM or FEM), the validation datasets (ranging from simulated signals to clinical epilepsy recordings), and the quantitative performance metrics reported in each study.

Method	Training data	Validation data	Reported performance	Reference
LSTM	Simulated scalp EEG using FEM (15 000 1s samples)	• Simulated data	• Mean localization error: 4.22 mm	[276]

ConvDip (CNN)	Simulated EEG with 3-shell BEM (100 000 1s samples)	• Simulated data • Human scalp EEG (perception task)	• Localization error: 11.05 mm, MSE: $3.9 \times 10^{-19} \mathrm{V}^2$ • Overlap with brain regions previously associated with perception	[277]

DeepSIF (ResBlock-LSTM)	Simulated sources from Jansen–Rit model + EEG via 3-shell BEM (620 256 1s samples)	• Simulated data • 3 public EEG EP datasets • 1 epilepsy iEEG dataset	• Localization error: 1.56 mm • Overlap with brain regions previously associated with the EPs • Epileptic foci localization (defined as the overlap with resection area) precision: 0.79, recall: 0.49	[45]

DMLN (VAE)	Simulated sources from Jansen–Rit model + EEG (24 000 10s samples)	• Public VEP EEG dataset • Public seizure EEG dataset	• Overlap with brain regions previously associated with the EPs • Epileptic foci localization precision: 0.91, recall: 0.81.	[278]

*Abbreviations:* **EEG** = electroencephalography; **iEEG** = intracranial electroencephalography; **LSTM** = long short-term memory; **CNN** = convolutional neural network; **ResBlock** = residual block; **VAE** = variational autoencoder; **FEM** = finite element method; **BEM** = boundary element method; **EP** = evoked potential; **VEP** = visual evoked potential; **MSE** = Mean Squared Error.

## 5. Clinical applications of neural source estimation

During recent decades, brain source modeling using EEG has become an active area of research with significant clinical applications [30, 283]. Noninvasive localization of active brain sources has been used to diagnose pathological, physiological, and functional abnormalities, including epilepsy, event-related potentials, and attention deficit/hyperactivity disorder (ADHD) [14]. This section summarizes clinical applications of source estimation and discusses future research directions.

### 5.1. Epileptogenic zone identification

Localizing the epileptogenic zone was a key motivation for brain source localization [14]. Among various neuroimaging modalities, EEG offers superior temporal resolution, making it particularly well-suited for investigating seizures and identifying their onset zones. However, unlike fMRI, EEG lacks high spatial resolution, which limits its ability to precisely localize neural sources [284]. Electroencephalographic source imaging (ESI) provides a noninvasive means of estimating the intracranial origins of epileptiform discharges based on scalp EEG recordings [285, 286]. Given that scalp EEG primarily detects discharges generated by spatially extended cortical sources, accurate source localization methods are crucial for delineating these spatial extents and supporting effective surgical planning [14].

As highlighted by Singh *et al*, ESI has evolved from theoretical biophysical modeling into a clinically viable tool, capable of improving diagnostic yield, particularly in patients with inconclusive MRI findings [41]. Methods such as minimum-norm family (e.g. MNE, wMNE, sLORETA, LAURA [287]) and high-resolution techniques like FINE [288] or beamformers enable reconstruction of both focal and network-level epileptogenic activity. These models rely on realistic head modeling, noise handling, and precise electrode localization to accurately capture the spatiotemporal dynamics of epileptic discharges. Several clinical studies, including Ding *et al* and Kim *et al*, have demonstrated the feasibility and utility of ESI in localizing the seizure onset zone, often with accuracy comparable to invasive modalities like PET and SPECT [286, 289]. Moreover, ESI has been adapt to sEEG to localize epileptogenic zone, wherein Vakilna *et al* demonstrated that resection of the estimated epileptogenic zone resection led to seizure freedom [290]. Collectively, these findings underscore the growing role of source imaging in presurgical workflows, particularly in guiding invasive planning and improving surgical outcomes.

### 5.2. Consciousness and anesthesiology

While scalp EEG has long been used in anesthesiology, combining it with source localization remains underutilized [291–294]. Study in [295] shows that (NMM-based tracking using the Jansen–Rit model and unscented Kalman filtering enables real-time estimation of anesthetic brain states from EEG while inferring underlying physiological dynamics during propofol-induced unconsciousness. Tapping into source-localized EEG enriched with physiologically grounded models could revolutionize anesthesia monitoring—by pinpointing not just when but where and why consciousness transitions occur in the brain.

### 5.3. Tumor detection

EEG source localization is increasingly recognized as a critical tool for applications involving the detection and monitoring of neurological abnormalities [14]. Although it is not currently a primary method for the initial detection of brain tumors, source localization may offer complementary insights by characterizing the functional impact of tumors on cortical activity. For instance, Selvam and Shenbagadevi [296] applied a modified wavelet-ICA technique to decompose scalp EEG signals into statistically independent components, which approximate underlying neural sources. While this approach does not constitute anatomical source localization in the traditional sense, it effectively enhances the functional separation of EEG signals, enabling tumor classification via a neural network. As more accurate and anatomically grounded source localization methods become available, future studies could explore their potential to augment existing diagnostic workflows, particularly when combined with high-resolution imaging modalities such as MRI or CT.

### 5.4. Closed loop deep brain stimulation

Deep Brain Stimulation is an effective treatment for motor symptoms in neurological disorders like Parkinson’s disease, but its success depends heavily on precise electrode placement. While imaging techniques like MRI and CT are limited by metal artifacts and safety risks, and microelectrode recording is accurate but invasive and time-consuming, EEG offers a noninvasive, portable, and low-cost alternative for functional brain mapping during or after surgery [297].

Recent studies have explored combining EEG with ESI to improve DBS localization. Notably, Iacono *et al* demonstrated the feasibility of localizing DBS electrodes by treating the DBS-induced EEG artifact as a signal [298]. Using a high-resolution head model and finite-difference time-domain simulations for the forward model, they applied the dSPM algorithm to solve the EEG inverse problem [298]. Their results showed that the DBS electrode location could be estimated with an accuracy of about 1.2–1.5 cm, supporting the potential for EEG-based, noninvasive intraoperative guidance [298]. Simplified linear inverse models have also been explored in the context of deep brain stimulation. For example, Chang *et al* used a geometry-based forward model combined with ICA to estimate local neural activity from multi-contact DBS electrodes. These estimates of population-level phase and amplitude were used to guide an adaptive closed-loop stimulation strategy. While not equivalent to full EEG source localization, this work demonstrates how inverse modeling can support real-time neuromodulation control [299].

#### 5.4.1. Additional applications of source estimation

EEG source imaging has also demonstrated significant potential across a range of neurodevelopmental and psychiatric disorders. For instance, ADHD subtypes can be differentiated based on distinct spatial patterns of brain activity, enabling more targeted and personalized diagnostics [300, 301]. In [300], hd-EEG combined with sLORETA source localization was used during a Go/Nogo task to investigate the effects of theta/beta neurofeedback in children with ADHD, revealing specific modulation of medial frontal response inhibition processes. A complementary study [301] further explored subtype-specific timing deficits in ADHD using similar techniques, identifying divergent neurophysiological mechanisms in inattentive and combined subtypes. Beyond ADHD, ESI has also been applied to investigate depressive rumination: a recent study [302] compared EEG activity during induced ruminative, neutral, and positive emotional states in a non-clinical population by utilizing ICA, DIPFIT2 source localization, and effective connectivity analysis. Additionally, ESI has been explored in a variety of other psychiatric and neurological conditions, including phobia, obsessive-compulsive disorder, diabetes-related cognitive impairment, restless leg syndrome, and the neurophysiological effects of psychiatric medications [14]. A systematic understanding of EEG source localization methodologies remains essential to enhance clinical utility, inform method selection, and guide future research and therapeutic strategies [14].

## 6. Existing tools for source estimation

### 6.1. Software

Several software packages are available to help researchers tackle inverse and forward modeling of neural sources. Some of these software are commercially available, with either FDA-clearance for clinical use in the United States or CE-marking for clinical use in the European Union [303–305]. An independent study found that the CURRY and BESA softwares had similar agreement to clinicians for locating the irritative zone of epilepsy, but neither software reliably identified the epileptogenic zone [306]. An overview of popular software is detailed in table 4. The open-source packages, particularly MNE-Python, Brainstorm, FieldTrip, and EEGLAB, have gained widespread adoption in the research community due to their flexibility, extensive documentation, and active user communities that continuously contribute new methods and improvements, although FieldTrip and EEGLAB generally require MATLAB licenses to run. While commercial software often provides streamlined workflows and dedicated technical support suitable for clinical environments, open-source alternatives offer greater transparency in implementation and the ability to customize algorithms for specific research questions. The choice between commercial and open-source solutions typically depends on the specific application context, regulatory requirements, available computational resources, and the level of methodological control needed by the user.

**Table 4.** Software for forward and inverse EEG/MEG modeling. This table categorizes widely used software packages, distinguishing between open-source community toolboxes and commercial platforms, including details details on the computing platforms, specific modeling capabilities, and regulatory clearances where applicable.

Software name	Platform	Description	Reference	Open source
Brainstorm	MATLAB (GUI-based)	User-friendly GUI for EEG/MEG analysis; supports multiple source estimation algorithms and forward models.	[195]	Yes
EEGLAB	MATLAB	Open-source toolbox for EEG analysis; supports plug-ins for source localization and connectivity.	[328]	Yes
FieldTrip	MATLAB	Flexible toolbox for EEG/MEG analysis with a focus on time-frequency and source analysis.	[329]	Yes
MNE-Python	Python	Open-source Python package for EEG/MEG data analysis; includes preprocessing, source localization, connectivity, and statistical analysis.	[330]	Yes
Open MEEG	Python/MATLAB	Forward problem solver for EEG/MEG using single/overlapping spheres and BEM.	[331]	Yes
DUNEuro	Python/MATLAB	Forward problem solver for EEG/MEG using FEM.	[332]	Yes
The Virtual Brain	Python	Large-scale brain network modeling platform; integrates structural and functional imaging data for neural mass model simulations.	[333]	Yes
SPM	MATLAB	Statistical Parametric Mapping software; supports EEG/MEG source reconstruction using Bayesian frameworks.	[334]	Yes
BESA	Windows	CE-marked commercial software for EEG/MEG source analysis, including individual FEM forward modeling, dipole modeling, distributed inverse solutions, and multimodal integration of MRI.	[305]	No
BrainVoyager	Windows/Linux/macOS	Primarily an fMRI analysis tool, but includes support for EEG/MEG integration and source localization.	[335]	No
CURRY	Windows	FDA-cleared and CE-marked commercial software for EEG/MEG source analysis, including individual BEM forward modeling, dipole modeling, distributed inverse solutions, and multimodal integration of MRI, PET, and SPECT.	[303]	No
GeoSource	macOS	FDA-cleared and CE-marked commercial software for source analysis of the company’s EEG system; research version includes individual FDM forward solver, dipole modeling, distributed inverse solution, and multimodal integration of MRI.	[304]	No
LORETA-KEY	Standalone (Windows)	Performs Low Resolution Brain Electromagnetic Tomography for EEG source localization.	[336]	No

*Abbreviations:* **EEG** = electroencephalography; **MEG** = magnetoencephalography; **GUI** = graphical user interface; **fMRI** = functional magnetic resonance imaging; **BEM** = boundary element method; **FEM** = finite element method; **FDM** = finite difference method; **PET** = positron emission tomography; **SPECT** = single-photon emission computed tomography; **FDA** = Food and Drug Administration; **CE** = Conformité Européenne (European Conformity).

### 6.2. Datasets

Open-source datasets are critical for advancing the field of neural source estimation. Neurophysiological data can be costly to acquire, and intracranial recordings are inherently invasive, limiting the available population pool. Further, storing the data can be challenging due to the size of multivariate, high-resolution, longitudinal recordings. Sharing this data also presents privacy concerns that must be addressed with informed consent procedures that protect the confidentiality of the patient or research subject.

Because of these inherent challenges, the number of available datasets is limited but rapidly growing. Simulated datasets, generated through (NMMs and forward modeling techniques, provide controlled environments for training supervised learning models and validating inverse modeling approaches. For real-world validation of source estimation and localization, some researchers have used intracranial stimulation [19, 298]. Other researchers have used clinically respected epileptogenic zones as the clinical ground truth of epileptic spike localization [45, 307]. Multimodal datasets are especially valuable by combining simultaneous recordings from noninvasive electromagnetic imaging (EEG, MEG, etc), hemodynamic responses (fNIRS, fMRI, etc), and invasive recordings (sEEG, ECoG, etc). These multimodal datasets enable more direct measurement of neural activity, which can validate existing source estimation methods. Further, these datasets provide ground truth measurements that may enable mapping noninvasive recordings to invasive recordings. Of these, we identified only two studies [19, 308] that contained synchronous EEG and sEEG recordings. Further work is needed to develop and validate models that estimate invasive recordings with noninvasive measurements.

Several established resources and repositories support open-access sharing of relevant neurophysiological data. These resources for neurophysiological data include, but are not limited to, the Data Archive for the BRAIN Initiative [309], EBRAINS [310], IEEG.org [311], OpenNeuro [312], and PhysioNet [313]. Other platforms to facilitate clinical and research data sharing that contain relevant data include Dryad [314], Figshare [315], Open Science Framework [316], the Radboud Data Repository [317], and Zenodo [318]. In addition to these platforms, the standardized brain imaging data structure (BIDS) and standardized formats for other neuroimaging modalities have increased the ease of using data collected from different institutions [319–324]. In table 5, we highlight several open-access datasets available to researchers.

**Table 5.** Open-access multimodal datasets with intracranial recordings. A selected list of publicly available datasets combining invasive and noninvasive modalities, enabling validation and development of inverse methods using ground-truth intracranial data across diverse tasks and patient groups.

Dataset	Sample size	Modalities	Task	Reference
ARC-COGITATE	*n* = 256 subjs; 576–1440 trials/subj; 0.5–1.5 s/trial	fMRI (*n* = 120), 306-sensor MEG + 64ch EEG (*n* = 102), iEEG (*n* = 34)	Visual stimuli to probe theories of consciousness (epilepsy patients)	[337]

Localize-MI	*n* = 7 subjs; Tot. 2393 epochs; 260 ms/epoch	256ch hd-EEG, MRI, sEEG electrical stimulation	Single pulse electrical stimulation and hd-EEG recording (epilepsy patients)	[19]

Open iEEG-fMRI	*n* = 51 subjs; 3 min. resting state; 6.5 min. movie stimulus	ECoG (*n* = 46), HD-ECoG (*n* = 6), sEEG (*n* = 16), fMRI (*n* = 51)	Audiovisual stimulus to probe perception and language comprehension (epilepsy patients)	[308]

B(RAIN)^2^	*n* = 75 subjs	ECoG, DBS locations, MRI	Synchronous deep brain stimulation and ECoG recording (PD patients)	[338]

sEEG-pupillometry	*n* = 10 subjs	128ch sEEG + Pupillometry	Memory tasks to measure neural activity and eye movements (epilepsy patients)	[339]

Mesoscale Insights	*n* = 5 subjs; 12 seizures	Multi-unit activity, sEEG, ECoG, Utah Microelectrode Array	Mesoscale seizure recordings (epilepsy patients)	[340]

s/EEG Verbal WM	*n* = 9 subjs; 50–350 trials/subj	sEEG, 10–20 scalp EEG	Verbal working memory task (epilepsy patients)	[341]

Multimodal movie	*n* = 16 subjs	Single-neuron, LFP, sEEG, fMRI, and eye-tracking	Eight min. movie excerpt (epilepsy patients)	[342]

fNIRs-EEG	*n* = 12 subjs	11–14ch fNIRS, 64ch EEG	Silently name and visualize objects (healthy participants)	[343]

EEG-MEG-fMRI	*n* = 19 subjs	Synchronous EEG & MEG, asynchronous fMRI	Perception task (healthy participants)	[344]

EEG-MFEIT	*n* = 23 subjs	Synchronous EEG & MF-EIT, CT, MRI	Multi-frequency electrical impedance tomography and scalp EEG in hyper acute stroke unit (stroke patients)	[127]

*Abbreviations:* **fMRI** = functional magnetic resonance imaging; **MEG** = magnetoencephalography; **EEG** = Electroencephalography; **iEEG** = intracranial electroencephalography; **hd-EEG** = high-density electroencephalography; **sEEG** = stereoelectroencephalography; **ECoG** = electrocorticography; **DBS** = deep brain stimulation; **PD** = Parkinson’s Disease; **WM** = working memory; **LFP** = local field potential; **fNIRS** = functional near-infrared spectroscopy; **MF-EIT** = multi-frequency electrical impedance tomography; **CT** = computed tomography; **MRI** = magnetic resonance imaging.

## 7. Roadmap for future research

In this section, we propose a strategic roadmap for the evolution of neural source estimation. As the field increasingly integrates advances in high-performance computing, multimodal imaging, and artificial intelligence, a structured approach will help coordinate efforts across disparate disciplines. The following framework delineates concrete milestones across 5 year, 10 year, and long-term horizons to help extend source localization from a specialized research technique into a robust, ubiquitous tool in neuroscience, brain-computer interfaces, and clinical workflows.

In the short-term (1–5 years), the field should prioritize the development of robust infrastructure across data, software, and hardware. The most critical milestone is the curation of large-scale, standardized, and diverse datasets, akin to ImageNet in computer vision [325] or the UK Biobank in genetics-informed drug discovery [326]. The data developed and published for neural source estimation should emphasize high-quality recordings and well-annotated labels such as task, stimuli, and/or diagnostic information, along with conformity to standardized formats such as BIDS while simultaneously adhering to privacy standards. The publication of additional datasets beyond those presented in table 5 will enable the rigorous validation of inverse algorithms against ground-truth data. To support this, software platforms such as Brainstorm and MNE-Python, which are already relatively more mature as shown in table 4, can evolve from analysis toolboxes into integrated pipelines capable of handling and automating the fusion of hd-EEG with complementary modalities such as OPM-MEG, fNIRS, and EIT. Simultaneously, hardware milestones can focus on reducing the cost of hd-EEG systems and validating the scalability of wearable sensors in ambulatory settings to move source estimation into ecological environments. In parallel, forward modeling during this short-term period can develop even more realistic head volumes leveraging modalities such as high resolution MRI and techniques like automated tissue segmentation algorithms to estimate conductivity and anisotropy for each subject. These advances should be complemented by openly shared forward-model benchmarks and reference pipelines to ensure reproducibility across laboratories. At the same time, inverse-model development can emphasize robustness by establishing shared benchmarks, standardizing metrics with uncertainty estimates, and ensuring methods remain reliable under noise and model mismatch. The research paradigms at this stage can focus across multiple levels: simulations, animal research, healthy volunteers, and clinical populations beyond epilepsy. Together, these short-term developments provide the essential computational and collaborative foundation for future development.

In the mid-term (5–10 years), the field can leverage this infrastructure to pursue algorithmic personalization, deeper multimodal integration, and more sophisticated modeling frameworks. Personalized forward models that incorporate subject-specific anatomy, conductivity, and diffusion-derived anisotropy could become widely adopted, enabling more accurate representations of individual biophysics. These personalized models will support the emergence of‘digital twin’ frameworks that blend physics-based simulations with data-driven components to capture non-linear, subject-specific neural dynamics [327]. Correspondingly, we expect inverse modeling to mature into hybrid architectures that integrate principled Bayesian or physics-based inference with deep learning modules capable of resolving complex, distributed sources while maintaining calibrated uncertainty estimates. During this period, multimodal source estimation should become routine, with standardized multimodal frameworks enabling more robust spatial and functional interpretability, and with low-latency inverse methods entering early-stage closed-loop BCI and clinical research applications.

In the long-term (10+ years), we anticipate that neural source estimation will extend its use beyond retrospective research toward a prospective clinical standard. Semi-automated, interpretable source-imaging systems should achieve multi-site validation and become integrated into diagnostic workflows across neurology and psychiatry, informing treatment planning and enabling more precise diagnostics and therapeutics such as neuromodulation and neurosurgery. Regulatory-ready software and procedural standards will support widespread deployment, while advances in real-time, personalized digital-twin models may allow source estimation to serve as the computational core of closed-loop BCIs and individualized therapies. In this long-term horizon, validated source estimates could function as primary, clinically actionable biomarkers used in routine care, fulfilling the vision of a mature and widely adopted neuroimaging technology.

## 8. Conclusion

Neural source estimation has evolved significantly with advancements in measurement techniques and computational models. From standard 10–20 EEG systems to more sophisticated modalities like hd-EEG, sEEG, MEG, and functional imaging techniques, continued advances in multimodal techniques have demonstrated improvements in the ability to localize and estimate brain activity more accurately. Forward models of neuron population activity, ranging from simple spherical models to complex FEM approaches, enable more precise simulations of how brain signals propagate, helping to constrain the inherently ill-posed inverse problem. Inverse modeling methods, including linear ECD reconstruction, current-density reconstruction, spatial filtering methods, have demonstrated clinical utility. Each approach relies on specific assumptions that suit different scenarios, such as a fixed number of focal sources versus distributed source patterns. Emerging nonlinear approaches, particularly those incorporating supervised machine learning, show promise in modeling signal propagation beyond the constraints of traditional biophysical volume conduction models. Since source estimation techniques were developed before the emergence of many powerful data-driven machine learning algorithms, future work can examine these nonlinear approaches to modeling source activity or even directly measure neural activity and map noninvasive measurements to these modalities. With continued innovation in theory, algorithms, and measurements, the future of neural source estimation holds promise for improved diagnostic and therapeutic strategies in neuroscience and neurology while opening the door to analyzing deep brain activity without performing surgery.

## Data Availability

No new data were created or analysed in this study.
